# A Bayesian Joint Model of Longitudinal Kidney Disease Progression, Recurrent Cardiovascular Events, and Terminal Event in Patients with Chronic Kidney Disease

**DOI:** 10.1007/s12561-024-09429-6

**Published:** 2024-05-09

**Authors:** Esra Kürüm, Brian Kwan, Qi Qian, Sudipto Banerjee, Connie M. Rhee, Danh V. Nguyen, Damla Şentürk

**Affiliations:** 1 Department of Statistics, University of California, Riverside, CA USA; 2 Department of Health Science, California State University, Long Beach, USA; 3 Department of Biostatistics, University of California, Los Angeles, CA USA; 4 Department of Medicine, University of California, Los Angeles, CA USA; 5 VA Greater Los Angeles Health Care System, Los Angeles, CA USA; 6 Department of Medicine, University of California, Irvine, CA USA

**Keywords:** End-stage kidney disease, Chronic kidney disease, Joint models, Recurrent events, Survival analysis

## Abstract

Nearly 15% (37 million) of adults in the United States (US) have chronic kidney disease (CKD). The longitudinal decline of kidney function is intricately related to the development of cardiovascular disease (CVD) and eventual “terminal” event (kidney failure and mortality) in patients with CKD. Understanding the mechanism and risk factors underlying the three key outcome processes, (1) CKD progression, (2) CVD, and (3) subsequent terminal event in the CKD patient population remains incomplete. Thus, in this work, we develop a novel trivariate joint model to study the risk factors associated with the interdependent outcomes of kidney function (as measured by longitudinal estimated glomerular filtration rate), recurrent cardiovascular events, and the terminal event. Efficient estimation and inference is proposed within a Bayesian framework using Markov Chain Monte Carlo and Bayesian P-splines for hazard functions. The proposed Bayesian framework is directly generalizable beyond trivariate outcome processes to accommodate other potential modeling of complex multi-disease processes. The method is applied to study the aforementioned trivariate processes using data from the Chronic Renal Insufficiency Cohort Study, an ongoing prospective cohort study, established by the National Institute of Diabetes and Digestive and Kidney Diseases to address the rising epidemic of CKD in the US.

**Supplementary Information:**

The online version contains supplementary material available at 10.1007/s12561-024-09429-6.

## Introduction

End-stage kidney disease (ESKD), the last stage of chronic kidney disease (CKD), requires dialysis or kidney transplantation to sustain life and affects over 809,000 individuals as of 2019 in the United States [1]. For individuals with ESKD, the prevalence of common cardiovascular disease (CVD) is extremely high at about 77% among hemodialysis patients and the most common causes of ESKD include diabetes and high blood pressure [1]. Non-dialysis dependent CKD is much more prevalent [1, 2] and the latest data (2015–2018) from the National Health and Nutrition Examination Survey (NHANES) estimate that 14.4% of adults in the US have CKD, defined by a low estimated glomerular filtration rate (eGFR $<60$ ml/min/1.73m^2^) or albuminuria (urine albumin to creatinine ratio $\ge 30$ mg/g). Understanding CKD progression, including the interdependent relationships with recurrent CV events and terminal event (e.g., ESKD or death), and the risk factors for each outcome (kidney function decline, recurrent CV events, and terminal event) in patients with CKD remains incomplete. Earlier studies have noted the association between even mild CKD and CVD [3].

To address this knowledge gap in risk factors that influence CKD and CVD progression, and morbidity and mortality, the National Institute of Diabetes and Digestive and Kidney Diseases (NIDDK) established the Chronic Renal Insufficiency Cohort (CRIC) Study in 2001 [4, 5]. The CRIC study is a currently ongoing prospective cohort study. Motivated by the broad aims of the CRIC study, we developed a novel Bayesian trivariate joint model to study the interdependent processes: (1) CKD progression defined by the longitudinal eGFR trajectories, which is a measure of kidney function, (2) recurrent CV events, and (3) the composite terminal event of ESKD or death (time-to-event). We note that ESKD/kidney failure is a common time-to-event outcome used in studies of kidney disease patients (e.g., see Yang et al. [6]) and although it is not a “terminal” event in the clinical sense, we treat it as so in the composite event definition because longitudinal follow-up of eGFR also ends at ESKD (defined as initiation of dialysis or kidney transplant).

Motivated by the goal of the CRIC study, we develop a novel Bayesian trivariate joint model to examine the effects of patient risk factors on the trivariate outcomes and to formally quantify the level of associations among the three outcomes. Of particular relevance to CKD patients and CV events, our proposed recurrent event submodel using gap times between recurrent CV events allows for modeling the varying effects of traditional primary risk factors (diabetes, hypertension, and history of CVD) on each CV event separately.

We note that there is an extensive literature on joint modeling of a longitudinal and time-to-event outcome as reviewed in Tsiatis and Davidian [7]. For joint models, a common approach to account for the dependency between two outcomes is through the use of a shared frailty/random effect as proposed in Wulfsohn and Tsiatis [8] and in joint modeling of longitudinal hospitalization and survival in ESKD patients (Kürüm et al. [9]) among others [10–15]. For recurrent event models, several main approaches have been used. The simplest is to include a frailty term to account for correlated events within subjects [16], although frailty models do not account for event dependence (e.g., prior events affect future events), which is particularly relevant for CVD since the occurrence of an event (e.g., myocardial infarction) can cause damage to the cardiovascular system. Others have proposed stratified variance-adjusted models to account for (a) within-subject correlation [17, 18] and (b) event dependence *indirectly* through stratified baseline hazards [19, 20]. To account for both (a) and (b), conditional frailty models, which incorporate a frailty term as well as baseline hazard stratification have been proposed for event gap time [21, 22], although stratification is inefficient [23]. Motivated by analysis of recurrent CV events, Lin, Luo and Davis [24] incorporated the aforementioned features by modeling event effects directly and allowing for event-varying covariate effects. Also, Ouyang et al. [25] proposed a Bayesian (bivariate) model for recurrent events (number of graft rejections after heart transplant) and death.

The literature on joint model of three processes, including recurrent event process to model recurrent events, is relatively limited [26–28, 30]. Of particular relevance to our work is the trivariate model proposed by Król et al. [28, 29] which was motivated to analyze jointly the outcomes of (1) tumor size, (2) occurrence of new lesions, and (3) death in patients with metastatic colorectal cancer using penalized maximum likelihood and cubic M-splines for the baseline hazard functions for recurrent and terminal event processes [28]. There are two distinctions between our trivariate model and that of Król et al. The first is that we are proposing a novel Bayesian model and associated estimation and inference framework. The second, and more important, is the particular model for recurrent event process in our proposed trivariate model formulation which utilizes *gap times* between recurrent events. This important aspect of the modeling choice is motivated by known clinical features of recurrent cardiovascular events: (a) occurrence of a CV event is highly connected to subsequent events (event effect) and (b) the effect of a risk factor may vary with each CV event (event-varying effects). We note that the work of Król et al. [28] allows for a more general form/function of the longitudinal process to be associated with the recurrent and terminal processes. Adapting such a general association structure between longitudinal and recurrent events and between longitudinal and terminal event is not directly feasible in our trivariate model because the submodels do not share the same time index. That is, while the longitudinal and survival submodels use time since initiation of the study as the time index, the recurrent events submodel is built using aforementioned gap times between recurrent events. The recurrent events modeling via gap times enables incorporation of differential event effects in addition to accommodating event-varying covariate effects which allow for potential varying effects of covariates on each CV event separately. Another trivariate model relevant to our current work is the Bayesian multivariate frailty model for (two) recurrent events processes and a terminal event [30] which was motivated by modeling of the time from tumor surgical resection to local recurrence (first recurrent event process), time from tumor surgical resection to distant recurrence (second recurrent event process), and time from surgical resection of the tumor to death. Although our proposed trivariate model is also within a Bayesian framework, it is distinct from [30] in that that three submodels do not share the same time index and, furthermore, the recurrent event submodel which incorporates the aforementioned features (event effects and event-varying effects of risk factors).

**Fig. 1:**
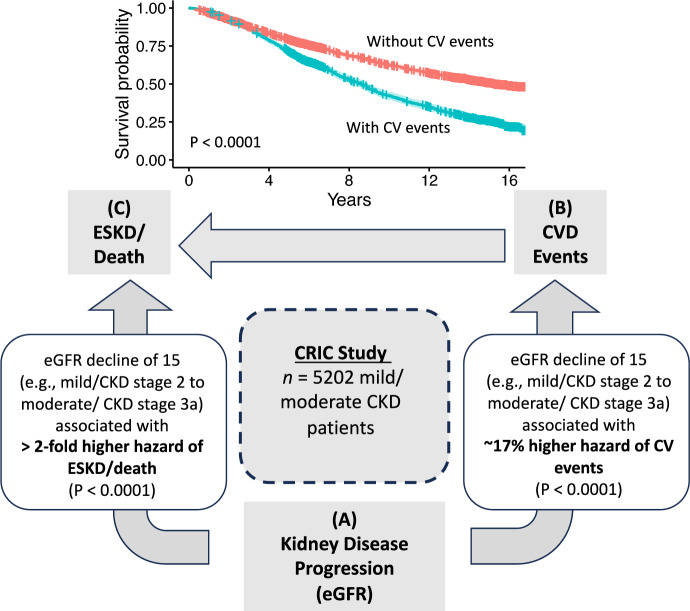
Exploratory analysis of the interdependencies among trivariate outcomes of (A) chronic kidney disease (CKD) progression as measured by longitudinal estimated glomerular filtration rate (eGFR); (B) cardiovascular disease (CVD) events; and (C) end-stage kidney disease (ESKD) or death (terminal event) for CKD patients in the Chronic Renal Insufficiency Cohort (CRIC) Study. Based on the CRIC data, an eGFR decline of 15 (ml/min/1.73 m^2^), approximately a change from CKD stage 2 (mild) to CKD stage 3a (early moderate) is associated with 17% higher hazard of CV events and a 2.1-fold higher hazard of kidney failure/death. Survival (time to terminal event) is significantly reduced for those with CV events compared to those without (Kaplan–Meier plot)

We also note that although the interdependencies among CKD progression, CVD and death/ESKD are established in the clinical literature [3, 4] and updated annually in the national report by the NIDDK-USRDS [1], we further motivate the need for joint trivariate modeling of these three key outcomes based directly on the CRIC Study data (consisting of $n=5202$ mild/moderate CKD patients followed for median of about 11.9 years). This is summarized and conceptualized in Fig. 1 which shows through exploratory analysis that an eGFR decline of 15 (ml/min/1.73 m^2^), approximately a change from CKD stage 2 (mild) to CKD stage 3a (early moderate) is associated with 17% higher hazard of CV events and a 2.1-fold higher hazard of kidney failure/death. Furthermore, survival (time to terminal event) is significantly reduced for those with CV events compared to those without (Fig. 1, Kaplan–Meier plot). Thus, modeling of the trivariate outcomes jointly is needed to not only provide estimation and inference for risk factors on outcomes jointly, but also to quantify the relative strength of associations/links among the three outcome processes.

The remainder of this paper is organized as follows. Details of the proposed Bayesian trivariate joint model are provided in Sect. 2, including the estimation and inference procedure. We present a novel analysis of kidney function decline, recurrent CV events, and terminal event processes using the CRIC Study data in Sect. 3. Section 4 summarizes efficacy of estimation via simulation studies and we conclude with a brief discussion in Sect. 5 of key findings and potential future works.

## Proposed Bayesian Joint Model of Trivariate Processes

### Model Specification

Let $Y_{i}(t)$ represent the longitudinal outcome (eGFR trajectory in our data application) for the *i*th subject at time *t* with $i=1, \ldots , n$. For the terminal event (ESKD or death), the true and observed event times are denoted by $T^{*}_{i}$ and $T_{i}$, respectively, for subject *i*. The observed terminal event time is defined as the minimum of the potential censoring time $C_{i}$ and $T_{i}^{*}$ with the event indicator defined as $\delta _{i}= I (T_{i}^* \le C_{i})$, where $I (\cdot )$ denotes the indicator function. For the recurrent event process (CV events), let $G^*_{ij}$ denote the *j*th true gap time (time between events $j-1$ and *j*), $R_{ij} = \sum _{d=1}^j G_{id}$ be the total time from the study onset to event *j*, and $C_{ij} = C_i - R_{i, j-1}$ be the *j*th gap censoring time with $j=1,\ldots , r_i$ (and $r_i$ is the total number of recurrent events before censoring). We denote the observed gap time and recurrent event indicator as $G_{ij} = \min (G_{ij}^*, C_{ij})$ and $\lambda _{ij}= I (G_{ij}^* \le C_{ij})$, respectively. To analyze the three outcome processes jointly, we propose the following trivariate model for longitudinal ($Y_{i}(t)$), recurrent ($r_{ij} (t \mid \cdot )$), and terminal event ($h_{i} (t \mid \cdot )$) outcomes:

$$\begin{aligned} Y_{i}(t)= & {} {\textbf{X}}_{i}^\textrm{T}\varvec{\beta }_l +{\textbf{Z}}_{i}^\textrm{T}\varvec{\phi }_{l} + \gamma t+ b_{i0} + b_{i1} t + \varepsilon _i(t), \\ r_{ij} (t \mid {\textbf{X}}_{i}, {\textbf{Z}}_{i}, {\textbf{b}}_i, \nu _i)= & {} h_{r0}(t)\exp \left( {\textbf{X}}_{i}^\textrm{T}\varvec{\beta }_{r} + {\textbf{Z}}_{i}^\textrm{T}\varvec{\phi }_{rj} + \sum _{m=0}^{j-1} \alpha _m + \eta _{r0} b_{i0} +\eta _{r1}b_{i1} + \nu _i\right) , \\ h_{i} (t \mid {\textbf{X}}_{i}, {\textbf{Z}}_{i}, {\textbf{b}}_i, \nu _i)= & {} h_{t0}(t)\exp ( {\textbf{X}}_{i}^\textrm{T}\varvec{\beta }_t + {\textbf{Z}}_{i}^\textrm{T}\varvec{\phi }_{t} +\eta _{t0} b_{i0} +\eta _{t1}b_{i1} + \zeta \nu _i), \end{aligned}$$

where, for ease of exposition, we separate secondary covariates (risk factors) and primary covariates (primary “exposure” variables or main risk factors) as ${\textbf{X}}_{i} = (X_{i1}, \ldots , X_{ip})^\textrm{T}$ and ${\textbf{Z}}_i = (Z_{i1}, \ldots , Z_{iq})^\textrm{T}$, respectively. The corresponding coefficients are denoted $\varvec{\beta }= (\varvec{\beta }_{l}^\textrm{T}, \varvec{\beta }_{r}^\textrm{T}, \varvec{\beta }_{t}^\textrm{T})$ and $\varvec{\phi }= (\varvec{\phi }_{l}^\textrm{T}, \varvec{\phi }_{r}^\textrm{T}, \varvec{\phi }_t^\textrm{T})$, respectively, in each submodel, and note that $\varvec{\phi }_{r} = (\varvec{\phi }_{r1}^\textrm{T}, \ldots , \varvec{\phi }_{rJ}^\textrm{T})^\textrm{T}$ is the event-varying coefficient vector in the recurrent event submodel. For our data analysis (Sect. 3), the main covariates (${\textbf{Z}}_i$) of focus are diabetes, hypertension and history of CVD and the recurrent event submodel captures the extent to which these main risk factors affect the onset of first, second, and subsequent CV events in CKD patients. Furthermore, the parameter $\alpha _m$ is the additional risk (hazard) introduced by the *m*th event ($\alpha _0 = 0)$, the vector $\varvec{\alpha }= (\alpha _1, \ldots , \alpha _{J-1})$ is the event effect, and *J* is the maximum number of recurrent events among all subjects. Note that $\exp (\alpha _m)$ is the hazard ratio of having an event (e.g., next CV event) for a patient with *m* previous events relative to a patient with $m-1$ previous events.

The association or link parameters among the three outcomes are $\eta _{*0}$, $\eta _{*1}$ ($*$ denoting *r* [recurrent] or *t* [terminal]), and $\zeta$. In particular, $\eta _{t0}$ and $\eta _{t1}$ measure the association between the longitudinal and the terminal outcome; $\eta _{r0}$ and $\eta _{r1}$ measure the association between the longitudinal and recurrent events; and $\zeta$ captures the relationship between the recurrent events and the terminal event. Also, ${\textbf{b}}_i = (b_{i0}, b_{i1})^\textrm{T}$ is the random effects (REs) vector with $b_{i0}$ and $b_{i1}$ as the random intercept and slope terms, respectively, $\nu _i$ is the fraility term, and $h_{*0}(t)$ ($*$ denoting *r* or *t*) is the baseline hazard function for recurrent and terminal process, respectively. The error terms $\varepsilon _i(t)$ are independent and follow a normal distribution: $\varepsilon _i(t)\sim {N}(0, \sigma _{\varepsilon }^2)$.

Let ${\textbf{u}}_i = ({\textbf{b}}_i^\textrm{T}, \nu _i)^\textrm{T}$ be the vector of random effects and is assumed to follow a multivariate normal distribution $N({\textbf{0}}, \varvec{\Sigma })$ such that $$\varvec{\Sigma }= \left( \begin{array}{lr} \varvec{\Sigma }_b &{} {\textbf{0}} \\ {\textbf{0}} &{}\sigma _\nu ^2 \end{array} \right)$$ with $$\varvec{\Sigma }_b = \left( \begin{array}{lr} \sigma _{b_0}^2 &{} \sigma _{01} \\ \sigma _{01} &{}\sigma _{b_1}^2 \end{array} \right)$$, $\sigma _{01} = \rho _b \sigma _{b_0} \sigma _{b_1}$, and $\rho _b$ is the correlation between the random intercept and slope terms. In our approach, the longitudinal outcome is linked to the recurrent and terminal outcome processes by the random effects ${\textbf{b}}_i$. The frailty term $\nu _i$ links the recurrent event and terminal event outcomes. The random effects ${\textbf{b}}_i$ and $\nu _i$ are assumed to be independent. Although we make distributional assumptions about the REs, empirical results show that the parameter estimation and inference in joint modeling are robust to these specifications, similar to standard joint models [11, 14, 15]. Note that, in our framework, longitudinal measurements and the recurrent events can be observed at the same time, however, neither can be observed after the terminal event time. This is the case for the CRIC Study, where eGFR measurement ends at ESKD (not meaningful after ESKD) and obviously at death.

### Estimation and Inference

To estimate the parameters in our joint modeling framework, we propose a Bayesian estimation procedure and derive posterior inferences using a Markov Chain Monte Carlo (MCMC) algorithm. Let $\varvec{\theta }= (\varvec{\beta }, \varvec{\phi }, \gamma , \varvec{\alpha },\varvec{\eta }, \zeta , \varvec{\sigma }^2, \rho _b, \varvec{\theta }_{h_0})^\textrm{T}$ be the parameters in all submodels with $\varvec{\beta }= (\varvec{\beta }_{l}^\textrm{T}, \varvec{\beta }_{r}^\textrm{T}, \varvec{\beta }_{t}^\textrm{T})$, $\varvec{\phi }= (\varvec{\phi }_{l}^\textrm{T}, \varvec{\phi }_{r}^\textrm{T}, \varvec{\phi }_t^\textrm{T})$, $\varvec{\alpha }= (\alpha _1, \ldots , \alpha _{J-1})$, $\varvec{\eta }= (\eta _{r0}, \eta _{r1}, \eta _{t0}, \eta _{t1})$, $\varvec{\sigma }^2 = (\sigma _{b_0}^2, \sigma _{b_1}^2, \sigma _\varepsilon ^2,\sigma _\nu ^2)$, and $\varvec{\theta }_{h_0}$ denoting the coefficients used to model the baseline hazard functions. The joint likelihood is obtained under the conditional independence assumption, that is, the random effects (REs: ${\textbf{u}}_i = ({\textbf{b}}_i^\textrm{T}, \nu _i)^\textrm{T}$) account for the association among the three outcomes, and given the REs, the outcomes are independent leading to the conditional likelihood

$$\begin{aligned} p({\textbf{Y}}_{i}, {\textbf{G}}_{i}, \varvec{\lambda }_i, T_i, \delta _{i} \mid {\textbf{u}}_i;\varvec{\theta })= & {} p( {\textbf{Y}}_{i} \mid {\textbf{b}}_{i}; \varvec{\theta }) \,\, p({\textbf{G}}_{i}, \varvec{\lambda }_{i} \mid {\textbf{u}}_i; \varvec{\theta })\,\, p(T_{i}, \delta _{i} \mid {\textbf{u}}_i; \varvec{\theta }) \nonumber \\= & {} \prod _{k=1}^{n_{i}} p (Y_{ik} \mid {\textbf{b}}_{i};\varvec{\theta }) \prod _{j=1}^{r_{i}} p(G_{ij}, \lambda _{ij} \mid {\textbf{u}}_i; \varvec{\theta }) p(T_{i}, \delta _{i} \mid {\textbf{u}}_i; \varvec{\theta }), \end{aligned}$$

where for the *i*th patient, ${\textbf{Y}}_{i}=(Y_{i1}, \ldots , Y_{in_{i}})^\textrm{T}$ denotes the $n_{i}\times 1$ vector of longitudinal outcomes with $Y_{ik} = Y_{ik}(t_{ik})$, $k=1, \ldots , n_{i}$, and ${\textbf{G}}_{i} = (G_{i1}, \ldots , G_{ir_{i}})^\textrm{T}$ and $\varvec{\lambda }_i = (\lambda _{i1}, \ldots , \lambda _{ir_{i}})^\textrm{T}$ are the $r_{i}\times 1$ vectors of recurrent event times and event indicators, respectively. Thus, the posterior distribution is obtained as

1 $$\begin{aligned} p(\varvec{\theta }, {\textbf{u}}_i \mid {\textbf{Y}}_{i}, {\textbf{G}}_{i}, \varvec{\lambda }_i, T_i, \delta _{i})\propto & {} p({\textbf{Y}}_{i}, {\textbf{G}}_{i}, \varvec{\lambda }_i, T_i, \delta _{i}\mid {\textbf{u}}_i,\varvec{\theta }) p({\textbf{u}}_i, \varvec{\theta }) \nonumber \\\propto & {} \prod _{k=1}^{n_{i}} p (Y_{ik} \mid {\textbf{b}}_{i},\varvec{\theta }) \prod _{j=1}^{r_{i}} p(G_{ij}, \lambda _{ij} \mid {\textbf{u}}_i, \varvec{\theta })\nonumber \\{} & {} p(T_{i}, \delta _{i} \mid {\textbf{u}}_i, \varvec{\theta }) p({\textbf{u}}_i\mid \varvec{\theta }) p(\varvec{\theta }). \end{aligned}$$

In our data application, where the longitudinal outcome is continuous, the likelihood contributions from each submodel is given by

2 $$\begin{aligned} p({\textbf{Y}}_{i} \mid {\textbf{b}}_i, \varvec{\theta })= & {} \prod _{k=1}^{n_{i}} \dfrac{1}{\sqrt{2\pi \sigma _{\varepsilon }^2}} \exp \left[ \dfrac{ \lbrace Y_{ik} - ( {\textbf{X}}_{i}^\textrm{T}\varvec{\beta }_l +{\textbf{Z}}_{i}^\textrm{T}\varvec{\phi }_{l} + \gamma t_{ik}+ b_{i0} + b_{i1} t_{ik} )\rbrace ^2}{2 \sigma _{\varepsilon }^2} \right] \nonumber \\ p({\textbf{G}}_{i}, \varvec{\lambda }_{i} \mid {\textbf{u}}_i, \varvec{\theta })= & {} \prod _{j=1}^{r_{i}} \Bigg[ \Bigg\{ h_{0r}(G_{ij}) \exp \Bigg( {\textbf{X}}_{i}^\textrm{T}\varvec{\beta }_r + {\textbf{Z}}_{i}^\textrm{T}\varvec{\phi }_{rj}  \nonumber \\{} & {}   +\sum _{m=0}^{j-1} \alpha _m +\eta _{r0} b_{i0} +\eta _{r1}b_{i1} + \nu _i \Bigg) \Bigg\} ^{\lambda _{ij}} \nonumber \\{} & {} \times \exp \Bigg\{ -\int _{0}^{G_{ij}} h_{0r}(s) \exp \Bigg( {\textbf{X}}_{i}^\textrm{T}\varvec{\beta }_r +{\textbf{Z}}_{i}^\textrm{T}\varvec{\phi }_{rj} \nonumber \\{} & {}  + \sum _{m=0}^{j-1} \alpha _m + \eta _{r0} b_{i0} +\eta _{r1}b_{i1} + \nu _i\Bigg) ds \Bigg\} \Bigg], \end{aligned}$$

and

$$\begin{aligned} p(T_{i}, \delta _{i} \mid {\textbf{u}}_i, \varvec{\theta })= & {} \left\{ h_{0t}(T_{i}) \exp ({\textbf{X}}_{i}^\textrm{T}\varvec{\beta }_t + {\textbf{Z}}_{i}^\textrm{T}\varvec{\phi }_{t} +\eta _{t0} b_{i0} +\eta _{t1}b_{i1} + \zeta \nu _i )\right\} ^{\delta _{i}} \\{} & {} \times \exp \left[ -\int _{0}^{T_{i}} h_{0t}(s) \exp ({\textbf{X}}_{i}^\textrm{T}\varvec{\beta }_t + {\textbf{Z}}_{i}^\textrm{T}\varvec{\phi }_{t} + \eta _{t0} b_{i0} +\eta _{t1}b_{i1} + \zeta \nu _i ) \textrm{d}s \right] . \end{aligned}$$

The integrals in the recurrent and terminal event submodels do not have closed-form solutions; hence, a numerical approximation is employed. We use the Gauss–Kronrod method.

We utilize Bayesian P-splines in estimation of the baseline hazard functions. In this approach, we use a relatively large number of equally spaced knots, and to avoid overfitting and obtain sufficiently smooth fitted curves, we apply a roughness penalty [31]. Specifically, $\log \lbrace h_{*0}(t) \rbrace = \sum _{w=1}^{\mathcal {W}} \psi _{h_{*0}, \omega } B_\omega (t)$ with $B_\omega (t)$ is the $\omega$th basis function of a B-spline, $\varvec{\psi }_{h_{*0}}$ denoting the corresponding ${\mathcal {W}}$-dimensional vector of spline coefficients ($*$ denoting *r* or *t*). Note that the proposed estimation and inference procedures can also accommodate parametric and other nonparametric forms for the baseline hazard function.

For the prior distributions, we use the normal distribution with mean zero and variance $\sigma ^2_\star$ for fixed-effects parameters $\varvec{\beta }= (\varvec{\beta }_{l}^\textrm{T}, \varvec{\beta }_{r}^\textrm{T}, \varvec{\beta }_{t}^\textrm{T})$, $\varvec{\phi }= (\varvec{\phi }_{l}^\textrm{T}, \varvec{\phi }_{r}^\textrm{T}, \varvec{\phi }_t^\textrm{T})$, $\gamma$ and $\varvec{\alpha }= (\alpha _1, \ldots , \alpha _{J-1})$, and association parameters $\varvec{\eta }= (\eta _{r0}, \eta _{r1}, \eta _{t0}, \eta _{t1})$ and $\zeta$. The hyperparameters are set as inverse gamma (IG): $\sigma ^2_\star \sim {IG}(a_\star , b_\star )$. For the variance terms, $\varvec{\sigma }^2 = (\sigma _{b_0}^2, \sigma _{b_1}^2, \sigma _\varepsilon ^2,\sigma _\nu ^2)$, we assume IG priors, $\sigma ^2_{*} \sim IG (a_{1*}, a_{2*})$ ($*$ denoting $b_0$, $b_1$, $\varepsilon$ or $\nu$). We utilize a positive uniform prior for $\rho _b$, the correlation between the REs in the longitudinal submodel ${\textbf{b}}_i = (b_{i0}, b_{i1})^\textrm{T}$, based on preliminary analysis/knowledge that subject-specific eGFR intercept and slope is positively correlated. In the estimation of the baseline hazard functions, we assign a normal distribution to the spline coefficients such that $\varvec{\psi }_{h_{*0}} | \kappa _{h_{*0}} \sim N(0, \kappa _{h_{*0}}\varvec{{\mathcal {P}}}_{h_{*0}})$ with the penalty matrix $\varvec{{\mathcal {P}}}_{*}$($*$ denoting *r* or *t*). The penalty matrix is calculated using the *v*th order difference matrix $\varvec{{\mathcal {D}}_v}$ of dimension ${\mathcal {W}} \times {\mathcal {W}}$, $\varvec{{\mathcal {P}}}_{*} = \varvec{{\mathcal {D}}_v}^\textrm{T}\varvec{{\mathcal {D}}_v} +10^{-4} {\textbf{I}}$, where ${\textbf{I}}$ denotes the ${\mathcal {W}} \times {\mathcal {W}}$ identity matrix. In applications, we utilize the commonly used second order difference matrix [31]. Similar to the variance terms, the variance parameter $\kappa _{h_{*0}}$, which controls the smoothness of the baseline hazard functions, is assigned IG priors, $\kappa _{h_{*0}} \sim IG (a_{1\kappa _{*}}, a_{2\kappa _{*}})$.

For inference on the parameters, we use credible intervals. Let $\tau$ denote a single parameter (any parameter within the full parameter vector $\varvec{\theta }= (\varvec{\beta }, \varvec{\phi }, \gamma , \varvec{\alpha },\varvec{\eta }, \zeta , \varvec{\sigma }, \rho _b, \varvec{\theta }_{h_0})^\textrm{T})$, and ${\hat{\tau }}$ and $\text {SD}(\tau )$ denote the mean and standard deviation of $\tau$ obtained based on a total of *L* MCMC samples, respectively. Then the $(1-\alpha )$ credible interval is given by $\left[ {\hat{\tau }} \pm \Phi _{\alpha /2} \text {SD}(\tau )\right]$, where $\Phi _{\alpha /2}$ denotes the $100\times (1-\alpha /2)$-percentile of the standard normal distribution.

We note that for simplicity of exposition, the submodels described above included (1) a common set of covariates; however, the estimation and inference procedures can accommodate design vectors with different dimensionality and composition for each submodel separately; and (2) time-invariant covariates notation, but they can include both baseline and time-varying covariates. Also, in the estimation procedure, we assigned the same IG prior for the variance terms in $\varvec{\sigma }^2$, however, our method can accommodate different priors for each variance term if needed. Similarly, we used the same number of knots in estimation of both baseline hazard functions, although the estimation procedure can handle a different number of knots for each baseline hazard function. All computations were performed in R (version 4.0.2), and as there are no closed-form solutions for the posterior distributions, we fitted our model using the Bayesian software JAGS (version 4.3.0) via the rjags package [32]. A combination of Metropolis sampling, Gibbs sampling, and other MCMC algorithms were used as sampling techniques while fitting the models [33]. We note that under the Bayesian framework, the random effects are also considered model parameters and by loading the ‘glm’ module of JAGS, we updated both fixed and random effects in the same block. We provide more details on the implementation in Section 7 of the Supplementary Materials. R codes and documentation for fitting the proposed trivariate joint model are made publicly available at https://github.com/esrakurum/Bayes-Trivariate.

## Application to the CRIC Study Data

### Study Cohort, Outcomes, Risk Factors and Primary Exposures

The CRIC study is an ongoing, multicenter, prospective longitudinal cohort study that enrolled adults with mild to moderate CKD since 2001. The study cohort included 5625 patients. The final analysis cohort included 5206 patients without missing data (excluded 7.4% due primarily to missing laboratory data).

Median follow-up time for study individuals was 11.9 years with maximum follow-up of about 16.9 years. The trivariate outcomes in our joint model analysis are (1) longitudinal eGFR progression (kidney function), (2) recurrent CV events, and (3) time to terminal event (defined as kidney failure or death, which are primary terminal events in the CKD population). CV events include congestive heart failure, myocardial infarction, cerebrovascular accident, and peripheral arterial disease events (e.g., angioplasty, bypass, surgical, amputation etc.), and atrial fibrillation. For recurrent CV events, we focus on the first three CV events which are typically of most clinical relevance and also which accounts for 80% of all CV events in the study cohort. The terminal event rate was 46.4% for the study cohort.

Risk factor domains for the trivariate outcomes include the following: demographic (age, sex, race); behavioral (current smoker, body mass index [kg/m^2^]); cardiovascular factors (systolic and diastolic blood pressure [SBP/DBP], high BP (BP > 130/80 mmHg), angle-brachial index [ABI]), laboratory measures (hemoglobin A1c [HbA1c], calcium, creatinine, glucose), use of angiotensin-converting enzyme (ACE) inhibitor or angiotensin receptor blocker (ARB) therapy, diabetes, hypertension, and history of CVD. Partly due to lack of novel methodology, how the main risk factors of hypertension, history of CVD, and diabetes jointly contributes to the trivariate outcomes have not been studied to date. Furthermore, understanding how these risk factors distinctly affect the occurrence of the first, second, and third CV events in patients contribute to risk reduction strategies since these CV events are major sources of morbidity and mortality in CKD patients. Therefore, our a priori specified primary (exposure) covariates are hypertension, history of CVD, and diabetes as these are known important contributing factors and causes of ESKD.

### Results

#### Cohort Characteristics

**Fig. 2:**
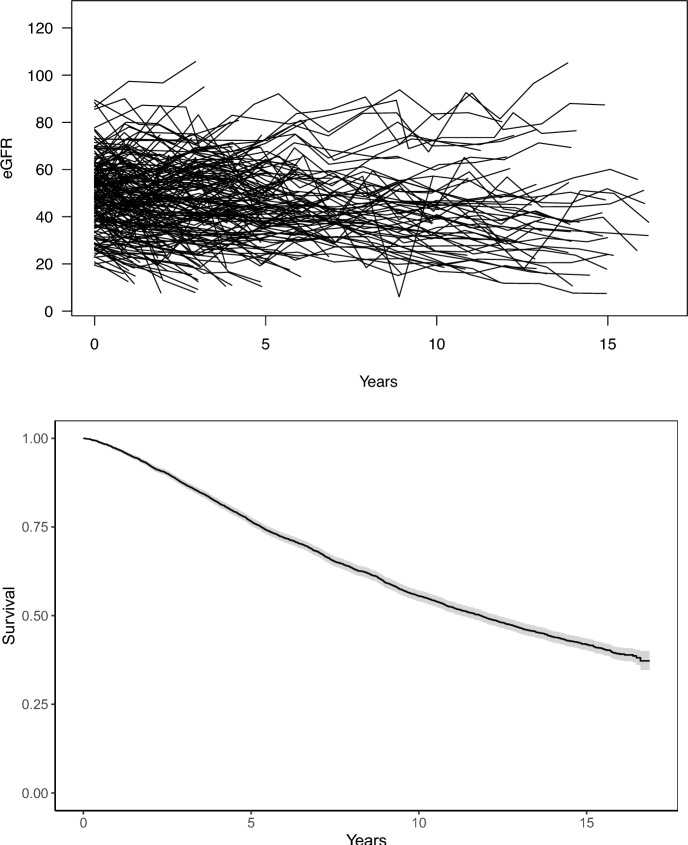
Longitudinal estimated glomerular filtration rate (eGFR) for 200 randomly selected individuals (top) and Kaplan–Meier survival curve for the study cohort with median survival of 11.7 years (bottom)

The mean baseline eGFR measurement was 48.4 ml/min/1.73m^2^ (standard deviation [SD] 15.6, median 48.2, Q1–Q3 [first–third quartile]: 37.1–58.8). Figure 2 (top) illustrates the longitudinal eGFR trajectories for 200 randomly selected individuals. Also, Fig. 2 (bottom) shows the survival outcome of the study cohort, which had a median survival of about 11.7 years and 75% had survival beyond five years. Patient baseline characteristics/risk factors are summarized in Table 1. The cohort consists of 43.4% females with patient age range 21–79 years (mean 59.6, SD 10.7). About 47% have high BP with mean SBP of 128 mmHg. The average BMI is about 32.2 (and about 56% of individuals have BMI $>30$) and about 12.5% are current smokers. With respect to the main risk factors, the cohort includes 51.1%, 86.5%, and 27.7% patients with diabetes, hypertension, and history of CVD, respectively. Other key characteristics include ABI <0.9 (15.1%), an indicator of peripheral artery disease; HbA1c (mean 6.7%); and calcium level (mean 9.3 g/dL). See Table 1 for details.

**Table 1:** Summary of patient risk factors

Variable	Mean (SD) or count (percent)^a^
Age (years)	59.58 (10.67)
Race/ethnicty	
Non-Hispanic White	2161 (41.51)
Non-Hispanic Black	2281 (43.81)
Other	764 (14.68)
Female	2257 (43.35)
Current smoker	649 (12.47)
Body mass index (kg/m^2^)	32.22 (7.52)
Angle-Brachial index < 0.9	787 (15.12)
ACE inhibitor/ARB therapy	3586 (68.88)
Estimated glomerular filtration rate, baseline (ml/min/1.73 m^2^)	48.38 (15.56)
Blood glucose (mg/dL)	119.78 (54.04)
Hemoglobin A1c (%)	6.67 (1.57)
Hemoglobin (g/dL)	12.68 (1.77)
Calcium (mg/dL)	9.25 (0.51)
Creatinine (Roche adjusted, mg/dL)	1.61 (0.55)
High blood pressure (BP > 130/80)	2463 (47.31)
Systolic blood pressure (mmHg)	128.24 (21.36)
Diastolic blood pressure (mmHg)	70.97 (12.59)
Primary risk factors	
Diabetes	2662 (51.13)
Hypertension	4501 (86.46)
History of cardiovascular disease	1444 (27.74)

^a^ For categorical variables; angiotensin-converting enzyme (ACE); angiotensin receptor blocker (ARB)

For the recurrent event analysis, the main interest is on elucidating whether the effects of these risk factors vary with each CV event in patients with CKD (i.e., whether the effects on the first CV event differ from the effects on subsequent CV events, for instance). In the study cohort, 69.4% of patients had no event and among 1594 individuals with one or more CV events, the majority (80%) of patients had 1 (795), 2 (311), or 3 (162) CV events.

#### Trivariate Joint Model: Risk Factors and Interrelationships Among Outcomes

The proposed Bayesian trivariate joint model of longitudinal eGFR, recurrent CV events, and the terminal event was fitted to the CRIC cohort of 5206 patients with mild to moderate CKD. The prior specifications used were the same as those described for the simulation studies in Sect. 4.2 below. We used three parallel chains with 10,000 iterations and with 3000 burn-in. Trace plots to assess satisfactory MCMC samples are provided in the Supplementary Table S2, S3, and S4 corresponding to the parameters of the longitudinal, recurrent, and terminal event submodel, respectively. In addition to examining the trace plots for convergence, we also monitored the scale reduction factor, *R*, to ensure that $R \sim 1$ as recommended by Gelman and Rubin [34].

Results of the Bayesian trivariate joint model are summarized in Table 2 (for longitudinal outcome) and Table 3 (for the recurrent and terminal event outcomes). Overall, longitudinal eGFR declined at a rate of $-$ 1.72 ml/min/1.73 m^2^ (95% credible interval (CI) $-$ 1.79 to $-$ 1.65) per year. Two primary risk factors were significantly associated with lower eGFR: hypertension ($-$ 1.5, CI $-$ 2.156 to $-$ 0.864) and history of CV disease ($-$ 0.483, CI $-$ 0.908 to $-$ 0.066). However, the third primary risk factor, diabetes, after adjusting for relevant lab markers (e.g., HbA1c) was not associated with eGFR. High BP ($-$ 0.64) and ACEI/ARB therapy ($-$ 1.24) were significantly associated with lower eGFR; see Table 2. Also, higher SBP, creatinine (Roche adjusted), and HbA1c were associated with lower eGFR, while higher calcium, glucose and hemoglobin were associated with higher eGFR. To interpret associations (effect sizes) across these lab measures with eGFR outcome, consider an increase of 50% of the standard deviation of each variable (SDs given in Table 1). For instance, an increase of 11 mmHg in SBP or 0.79% in HbA1c is associated with lower eGFR of $-$ 0.33 and $-$ 0.16, respectively. Also, female, higher age, and non-hispanic white were associated with lower eGFR. Details are summarized in Table 2. Subject-specific variation represents a substantial proportion of total variation in eGFR and intercept and slope random effects were positively correlated ($\rho _{01}$); see Table 4(ii).

**Table 2:** Results of trivariate joint model: Longitudinal outcome

Variable	(A) Longitudinal eGFR
	Estimate (95% CI)
Intercept	52.191 (51.452, 52.941)*
Time, $\gamma$	$-$1.723 ($-$1.793, $-$1.653)*
Age (years)	$-$0.306 ($-$0.327, $-$0.284)*
Black (ref. white)	6.242 (5.766, 6.740)*
Other	0.980 (0.368, 1.611)*
Female	$-$10.011 ($-$10.492, $-$9.528)*
Smoker	$-$0.354 ($-$0.983, 0.282)
Body mass index	0.016 ($-$0.013, 0.044)
ABI $< 0.9$	$-$0.022 ($-$0.608, 0.559)
ACEI/ARB use	$-$1.239 ($-$1.706, $-$0.802)*
Glucose (mg/dL)	0.007 (0.002, 0.012)*
Hemoglobin A1c (%)	$-$0.204 ($-$0.395, $-$0.003)*
Hemoglobin (g/dL)	0.163 (0.019, 0.301)*
Calcium (mg/dL)	1.010 (0.581, 1.414)*
Creatinine (mg/dL)	$-$25.632 ($-$26.072, $-$25.214)*
High BP	$-$0.636 ($-$1.203, $-$0.027)*
SBP (mmHg)	$-$0.03 ($-$0.045, $-$0.015)*
DPB (mmHg)	0.014 ($-$0.009, 0.037)
Primary risk factors	
Diabetes	0.072 ($-$0.587, 0.415)
Diabetes—$\phi _{11}$	–
Diabetes—$\phi _{12}$	–
Diabetes—$\phi _{13}$	–
Hypertension	$-$1.500 ($-$2.156, $-$0.864)*
Hypertension—$\phi _{21}$	–
Hypertension—$\phi _{22}$	–
Hypertension—$\phi _{23}$	–
CVD	$-$0.483 ($-$0.908, $-$0.066)*
CVD—$\phi _{31}$	–
CVD—$\phi _{32}$	–
CVD—$\phi _{33}$	–
$\alpha _1$	–
$\alpha _2$	–

Effects of patient risk factors on kidney disease progression measured by (A) longitudinal estimated glomerular filtration rate (eGFR). Effect sizes (estimates) are given for one unit change in covariates

*ACEI* angiotensin-converting enzyme inhibito, *ARB* angiotensin receptor blocker, *BP* blood pressure, *CVD* cardiovascular disease, *HbA1c* hemoglobin A1c, *SBP, DBP* systolic and diastolic BP

*95% credible interval (CI) does not include estimate of 0

**Table 3:** Results of trivariate joint model: Recurrent events and terminal event components

Variable	(B) Recurrent CV events	(C) Terminal event
	HR (95% CI)	HR (95% CI)
Intercept	–	–
Time, $\gamma$	–	–
Age (years)	1.034 (1.027, 1.040)*	1.028 (1.021, 1.036)*
Black (ref. white)	0.856 (0.764, 0.958)*	0.619 (0.536, 0.711)*
Other	0.814 (0.694, 0.953)*	1.009 (0.847, 1.203)
Female	0.960 (0.858, 1.074)	1.412 (1.228, 1.624)*
Smoker	1.296 (1.119, 1.492)*	1.508 (1.27, 1.788)*
Body Mass Index	1.015 (1.009, 1.022)*	1.001 (0.993, 1.01)
ABI $< 0.9$	1.346 (1.196, 1.511)*	1.332 (1.146, 1.547)*
ACEI/ARB use	0.944 (0.845, 1.055)	0.916 (0.799, 1.047)
Glucose (mg/dL)	1.000 (0.999, 1.001)	1.000 (0.998, 1.001)
Hemoglobin A1c (%)	1.115 (1.067, 1.163)*	1.093 (1.037, 1.153)*
Hemoglobin (g/dL)	0.961 (0.931, 0.993)*	0.918 (0.88, 0.954)*
Calcium (mg/dL)	0.824 (0.742, 0.908)*	0.793 (0.699, 0.895)*
Creatinine (mg/dL)	1.603 (1.446, 1.784)*	9.272 (7.973, 10.946)*
High BP	0.969 (0.838, 1.117)	0.985 (0.824, 1.171)
SBP (mmHg)	1.002 (0.999, 1.006)	1.008 (1.004, 1.013)*
DPB (mmHg)	0.998 (0.993, 1.003)	0.999 (0.993, 1.006)
Primary risk factors		
Diabetes	–	1.209 (1.036, 1.413)*
Diabetes—$\phi _{11}$	1.151 (1.000, 1.330)	–
Diabetes—$\phi _{12}$	0.969 (0.803, 1.172)	–
Diabetes—$\phi _{13}$	0.808 (0.643, 1.026)	–
Hypertension	–	1.315 (1.062, 1.644)*
Hypertension—$\phi _{21}$	1.586 (1.259, 2.004)*	–
Hypertension—$\phi _{22}$	1.179 (0.868, 1.621)	–
Hypertension—$\phi _{23}$	1.234 (0.800, 1.896)	–
CVD	–	1.822 (1.605, 2.071)*
CVD—$\phi _{31}$	2.643 (2.342, 2.986)*	–
CVD—$\phi _{32}$	1.718 (1.451, 2.040)*	–
CVD—$\phi _{33}$	1.675 (1.346, 2.088)*	–
$\alpha _1$	5.468 (3.755, 7.949)*	–
$\alpha _2$	1.597 (0.938, 2.713)	–

Effects of patient risk factors on (B) recurrent cardiovascular (CV) events, and (C) terminal event (kidney failure or death). Effect sizes (hazard ratios [HRs]) are given for one unit change in covariates

*ACEI* angiotensin-converting enzyme inhibitor, *ARB* angiotensin receptor blocker, *BP* blood pressure, *CVD* cardiovascular disease, *HbA1c* hemoglobin A1c, *SBP, DBP* systolic and diastolic BP

*95% credible interval (CI) does not include hazard ratio (HR) of 1

Individual variability explained by the random slope in the longitudinal eGFR trajectories was significantly associated with the composite terminal event (kidney failure or death): $\eta _{t1}=-0.698$ (CI $-$0.756 to $-$0.642). This strong association is expected since kidney function decline leads to eventual kidney failure or death (Table 4(i)). Variability in the random intercept was not associated with the terminal event ($\eta _{t1}=0.007$, 95% CI $-$0.006 to 0.022). Also, variability explained by the random slope in kidney progression was also significantly associated with recurrent CV events, although the estimated association/effects size was about 37% relative to the association with the terminal event ($\eta _{r1}=-0.256$, 95% CI $-$0.287 to $-$0.227). Although the association between CV events and variability in the random intercept was statistically significant, the relative effect size was small ($\eta _{r0}=0.021$, 95% CI 0.010 to 0.021). There was also strong positive association between CV events and kidney failure/death ($\zeta = 0.997$, 95% CI 0.808 to 1.226).

**Table 4:** (i) Estimates of associations/linkage among trivariate outcomes: (A) longitudinal estimated glomerular filtration rate (eGFR), (B) recurrent cardiovascular (CV) events, and (C) terminal event (kidney failure or death), and (ii) model variance components

	Estimate	95% CI
(i) Association/link parameters		
eGFR and recurrent CV events link		
$\eta _{r0}$	0.021	(0.010, 0.031)*
$\eta _{r1}$	$-$0.256	($-$0.287, $-$0.227)*
eGFR and terminal event link		
$\eta _{t0}$	0.007	($-$0.006, 0.022)
$\eta _{t1}$	$-$0.698	($-$0.756, $-$0.642)*
CV Events and terminal event link		
$\zeta$	0.997	(0.808, 1.226)*
(ii) Variance components		
Var(eGFR intercept), $\sigma ^2_{b0}$	35.002	(33.082, 37.030)*
Var(eGFR slope), $\sigma ^2_{b1}$	5.300	(4.963, 5.670)*
Correlation (intercept, slope), $\rho _{01}$	0.308	(0.300, 0.327)*
Measurement error variance, $\sigma ^2_{\epsilon }$	37.782	(37.140, 38.432)*
$\sigma ^2_{\nu }$ (terminal and recurrent link random effect)	0.567	(0.446, 0.692)*

*95% credible interval (CI) does not include estimate of 0

Next, we examined the event-varying effects of primary risk factors on CV events as well as event effects (effect of prior CV events on future CV events) for CKD patients from the recurrent event model results (Table 3(B)). Patients with a history of CVD (relative to patients without CVD) have the highest hazard ratio (HR) for the first CV event (HR 2.64, CI 2.34–2.99) and risk (hazard) of CV event continued to remain significantly high for the second (HR 1.72, CI 1.45–2.04) and third (HR 1.68, CI 1.35–2.09) CV event, although the risk was reduced by more than half compared to risk for the first CV event. Hypertension was associated with 59% higher hazard of the first CV event (HR 1.59, CI 1.26–2.00), but not associated with subsequent CV events. The effect of diabetes was not directly associated with the sequence of CV events, although potential effect of diabetes is through HbA1c control, where the hazard of a CV event is 12% higher for each 1% increase in HbA1c (HR 1.12, CI 1.07–1.16). Thus, diabetes was not associated with CV events, after accounting for effective disease control (reduction of HbA1c). Other modifiable risk factors associated with higher hazard of CV events include smoking, ABI $< 0.9$, higher BMI, and lower calcium level.

CV event effects are also summarized in Table 3(B) (${\widehat{\alpha }}$’s), which shows that the effect of each CV events varies. More specifically, for CKD patients, the hazard of the next CV event for a patient with 1 prior CV event is substantial relative to a patient without any CV event (HR 5.47, CI 3.75–7.95). However, for a patient with 2 past CV events, the hazard of the next (third) CV event is drastically reduced (and not significant): HR 1.60, CI 0.94-2.71.

With respect to risk factors for the terminal event (kidney failure or death), all three primary risk factors were associated with 21% to 82% higher hazard of terminal event (diabetes HR 1.21; hypertension HR 1.32; and CVD HR 1.82). See Table 3(C). In addition to these comorbidities, markers of disease management/control of hypertension and diabetes were also significantly associated with higher hazard of terminal event: higher risk of terminal event by 9% for 1% higher HbA1c, about 13% for 15 mmHg higher SBP, and 33% for ABI $<0.9$. Since the study cohort comprises of mild to moderate CKD, other important modifiable risk factors (which were found to be significant) include smoking and lower calcium level. Other factors associated with higher risk of terminal event include non-hispanic white race and female.

####  Comparison to Simple Analysis of Each Outcome Separately

Simpler analysis models for each outcome separately were also examined. Supplementary Table S9 (A-B-C) summarizes the analyses for the longitudinal eGFR, recurrent CV events, and terminal event. Overall, the effects of all covariates on kidney function (eGFR trajectories) from the simple model were similar to the joint trivariate model for the longitudinal component, including inference (significance of covariate effects; Table S9, column A). Of note, conclusions regarding the effects of primary exposures (diabetes, hypertension, and history of CVD) on kidney function remained the same, although we note that the decline in kidney function over time was attenuated in the simple model ($-$1.339, 95% CI $-$1.415 to $-$1.261 compared to $-$1.723, 95% CI $-$1.793 to $-$1.653).

Several aspects of the results for the simple analysis of terminal event alone were also overall similar to the trivariate model (Figure S9, column C). For example, inference based on CIs of the primary exposure variables (diabetes, hypertension and CVD) were consistent between the simple and trivariate analysis. However, compared to the joint trivariate model estimation, we note that the effect estimates for creatinine (HR 3.152, 95% CI 2.927–3.387) and history of CVD (HR 1.523, 95% CI 1.394–1.662) were substantially attenuated in the simple model. Furthermore, inferences regarding the effects of the demographic variables, including age (HR 1.002, 95% CI 0.997–1.007) and female (HR 1.038, 95% CI 0.094–1.143) were significant in the simple analysis.

The results for the simple analysis of recurrent CV events alone are given in Table S9 (column B). Here, we observe that although the overall conclusions regarding the primary exposure variables were similar (magnitude of estimates and inference), several differences were notable. The effects of several significant risk factors were smaller (e.g., creatinine HR 1.241 vs. 1.603) as well as conflicting inferences for race and female.

We note that these comparisons between the simple analyses and the trivariate model results are consistent with simulation studies, detailed in Section 4 (see also Supplementary Figure S1), which show that simple analysis ignoring the interdependent trivariate outcomes can lead to biased estimates and invalid inference. As detailed in the Supplementary Materials, invalid inference is worse for recurrent (and terminal event models) while the longitudinal outcome is least affected. (See Figure S1.) This is due to the fact that all model parameters are simultaneously estimated in the trivariate model framework and the association of the longitudinal outcome with recurrent and terminal (as well as the association between recurrent and terminal event) are accounted for in the estimation and inference procedure. Thus, simple analyses presented in Supplementary Table S9 should be interpreted with caution in light of potentially biased estimation and invalid inference.

####  Sensitivity Analyses: Hyperparameters and Number of Knots

As described earlier, the results of the CRIC data analysis uses hyperparameters such that $\sigma ^2_\star \sim \text {IG}(1, 0.005)$. We conducted sensitivity analyses with respect to hyperparameters, specifically using IG(1, 0.05) and IG(1, 0.5). The results are summarized in Supplementary Table S5, S6, and S7 corresponding to longitudinal, recurrent and terminal event submodels, respectively. The main results reported earlier largely demonstrated insensitivity to changes in the hyperparameter settings. Similarly, estimates of association/link parameters and variance components of the trivariate model were not sensitive to hyperparameter settings (Supplementary Table S8).

We note that with respect to the number of knots used for the baseline hazard, we followed the results and recommendations provided by Eilers and Marx, Lang and Brezger, and Eilers and Marx [31, 35, 36] and chose 20 equally-spaced knots while estimating the baseline hazard functions using P-splines. These authors demonstrated via extensive simulation studies that equally-spaced knots with a choice of a moderately large number of knots (usually taken to be 20) should be enough to ensure adequate flexibility for various different functional forms. In addition, under the P-splines, Ruppert [37] found that the number of knots is not a crucial parameter since the smoothing is controlled by the penalty. (Indeed, this was overall the case for our model when we examined various number of knots; results not shown.)

####  Model Fit

Finally, regarding the analysis of the CRIC data, we assessed relative model fit using the deviance information criterion (DIC). The criterion is a Bayesian measure of model fit and complexity proposed by Spiegelhalter et al. [38], where DIC $=$ ‘goodness of fit’ $+$ ‘complexity’ and, thus, penalizes for model complexity. (See also Gelman et al. [39].) To assess the relative fit of the trivariate model, we compared DIC for the trivariate model fit with four simpler models, including: (M0) a baseline “null” model that excludes all covariates; (M1) includes only linkages between longitudinal process with recurrent and with terminal event (excludes linkage between recurrent and terminal events); (M2) includes only linkages between longitudinal process and terminal event and between recurrent and terminal (excludes linkage between longitudinal and recurrent event); and (M3) includes only linkages between longitudinal process and recurrent event and between recurrent and terminal (excludes linkage between longitudinal and terminal event). We note that models M1, M2, and M3 each explores simpler “bivariate” linkages (associations) among the three outcomes. Further details and specification of these models can be found in Section 6 of the Supplementary Materials. Table 5 summarizes the penalized model fit via DIC. The results show that the DIC for the trivariate model is the smallest and, furthermore, the difference in DICs between each simpler model with the (full) trivariate model is substantial, clearly showing that the trivariate model provided the best fit accounting for model complexity.

**Table 5:** Bayesian model fit based on deviance information criterion (DIC) for the trivariate joint model of (L) longitudinal, (R) recurrent events, and (T) terminal event compared to a null model (M0: excludes covariates) and three bivariate association models (M1: includes L-R and L-T linkages; M2: includes L-T and R-T linkages; M3: includes L-R, R-T linkages)

Model	DIC	DIC difference with trivariate model
M0: “Null” model	2658899329	14050
M1: Bivariate linkages L-R and L-T	2658888108	2929
M2: Bivariate linkages L-T and R-T	2658887812	2533
M3: Bivariate linkages L-R, R-T	2658904822	19543
Trivariate L-R, L-T, and R-T	2658885279	–

## Simulation Studies

### Design

Simulation studies were conducted to evaluate the performance of estimation and inference for the proposed Bayesian trivariate model. Additionally, we evaluated the bias in estimation and impact on correct inference in simpler analysis models that ignore the trivariate joint outcomes structure, such as fitting models for each outcome individually or jointly modeling only two outcomes (e.g., ignoring the effect of recurrent events process on terminal event). These additional simulation studies are described in more details in the Supplementary Materials.

For the simulation studies, datasets were generated with similar characteristics to the CRIC study data. More specifically, each dataset was generated according to the following trivariate model.

- *Longitudinal process*: $Y_i(t) = \beta _{0} + X_{i} \beta _{l} + Z_{i} \phi _{l} + \gamma t + b_{i0} + b_{i1} t + \varepsilon _i(t)$, where $(\beta _0, \beta _l, \phi _l, \gamma ) = (47, {0.6}, -1.3, -1.5)$, random intercept and slope effects ${\textbf{b}}_i = (b_{i0}, b_{i1})^\textrm{T}\sim N( {\textbf{0}}, \varvec{\Sigma }_b)$ with $\varvec{\Sigma }_b = [\sigma _{b0}^2, \sigma _{01}; \sigma _{01}, \sigma _{b1}^2]$, $\sigma _{01} = \rho _b\sigma _{b0}\sigma _{b1}$, $\rho _{b} = 0.5$, $\sigma _{b0}^2 = 1.25$, and $\sigma _{b1}^2 = 0.80$, and the error $\varepsilon _i(t) \sim N(0, \sigma ^2_\varepsilon )$ with $\sigma ^2_\varepsilon = 1.32$. The random intercept had higher variance than slope and they are positively correlated, similar to observed random effects structure for longitudinal eGFR in the CRIC data.
- *Recurrent event process*: $r_{ij}(t \mid {\textbf{b}}_i, \nu _i) = h_{r0}(t) \exp ( X_{i} \beta _{r} + Z_{i} \phi _{rj} + \sum _{m=0}^{j-1} \alpha _{m} + \eta _{r0} b_{i0} + \eta _{r1} b_{i1} + \nu _i )$, where $\beta _r=0.2$, event-varying covariate effects $(\phi _1, \phi _2, \phi _3)=(1.04, 0.93, 0.79)$, event effects ($\alpha _1, \alpha _2) =(0.8, 0.53)$ with $\alpha _0 = 0$. The positive association between the longitudinal process and recurrent events was modeled through $\eta _{r0} = 0.6$ and $\eta _{r1} = 0.6$. The random effect $\nu _i \sim N(0, \sigma _\nu ^2)$ with $\sigma _\nu ^2 = 1.44$ links the recurrent event process to the terminal (survival) event process described next.
- *Terminal event process*: $h_{i}(t \mid {\textbf{b}}_i, \nu _i) = h_{t0}(t) \exp (X_{i} \beta _{t} + Z_{i} \phi _{t} + \eta _{t0} b_{i0} + \eta _{t1} b_{i1} + \zeta \nu _i )$, where $(\beta _t, \phi _t)=(0.5, 0.45)$. We mimic the CRIC data by setting a relatively stronger positive association between the terminal event and recurrent event with $\zeta = 1.2$. Association between the longitudinal process and terminal event is model via $\eta _{t0} = 0.9$ and $\eta _{t1} = 0.9$.

The baseline covariate $X_i$ was generated from a normal distribution with mean 1.5 and variance 1 and the event-varying covariate, $Z_i$, was generated as a binary variable with probability 0.6. For each subject, the longitudinal measurements were randomly selected on the interval [0, 1] before censoring by terminal event process. For the recurrent and terminal events, the true event times were simulated using the inverse probability integral transformation with Weibull baseline hazard functions $h_{r0}(t) = 0.0875t^{0.75}$ and $h_{t0}(t) = 0.035 t^{0.75}$, respectively. We present results from two censoring rates, 50% and 65%, with their corresponding recurrent event rates, 45% and 30%, respectively. We simulated 300 data sets for each sample size of $n=2000$ and $n=4000$.

### Simulation Results

For the proposed Bayesian estimation, we utilized a normal prior with mean zero and variance $\sigma ^2_\star$ for fixed-effects parameters $\varvec{\beta }= (\varvec{\beta }_{l}^\textrm{T}, \varvec{\beta }_{r}^\textrm{T}, \varvec{\beta }_{t}^\textrm{T})$, $\varvec{\phi }= (\varvec{\phi }_{l}^\textrm{T}, \varvec{\phi }_{r}^\textrm{T}, \varvec{\phi }_t^\textrm{T})$, $\gamma$ and $\varvec{\alpha }= (\alpha _1, \ldots , \alpha _{J-1})$, and association parameters $\varvec{\eta }= (\eta _{r0}, \eta _{r1}, \eta _{t0}, \eta _{t1})$ and $\zeta$. We used the hyperparameter $\sigma ^2_\star \sim IG(1, 0.005)$. For the variance terms, $\varvec{\sigma }^2 = (\sigma _{b_0}^2, \sigma _{b_1}^2, \sigma _\varepsilon ^2,\sigma _\nu ^2)$, we used IG priors with parameters $(a_{1*} = 0.001, a_{2*} =0.001)$ ($*$ denoting $b_0$, $b_1$, $\varepsilon$ or $\nu$). We used a uniform prior for the correlation between intercept and slope random effects: $\rho _b \sim \text {U}(0.3, 1)$. The baseline hazard functions $h_{0r}(t)$ and $h_{0t}(t)$ were estimated via the Bayesian P-splines with 20 equally-spaced knots and a second-order penalty. The priors $(a_{1\kappa _{*}} =1, a_{2\kappa _{*}}=0.005)$ were selected for the variance parameter $\kappa _{h_{*0}}$ ($*$ denoting *r* or *h*). We ran three parallel chains for each simulated data set with 12,000 iterations per chain, with the first 2000 iterations discarded as burn-in period. We set thinning to keep 2000 posterior samples in each chain, thus using 6000 samples for estimation and inference.

The performance of our proposed estimators was studied using the bias, mean-squared error (MSE), and coverage probabilities (CP) of 95% credible intervals based on 300 Monte Carlo runs at sample sizes $n=2000$ and 4000. Results for $n=2000$ with 50% and 65% censoring are summarized in Table 6. The estimation for $n=2000$ performed well with relatively low bias and MSE. For inference, the CP are reasonably close to 95% for longitudinal, recurrent events, and terminal event models, except for the linkage (between terminal and recurrent) parameter $\zeta$, where the coverage was lower at about 87%. This is not unexpected for this lower sample size setting and for a complex trivariate model with high level of censoring. As expected, the CP for all parameters, including the CP for $\zeta$ improved and were close to 95% under the higher sample size setting of $n=4000$ (Table 7). Estimation and inference performance was acceptable for all variance and correlation parameters.

**Table 6:** Simulation results for $n=2000$ and two censoring rates: 50% and 65%

		50% censoring			65% censoring		
		45% recurrent events			30% recurrent events		
	True	Bias	MSE	CP	Bias	MSE	CP
(A) Longitudinal							
$\beta _{0l}$	47.00	0.003	0.003	94.5	0.008	0.004	94.6
$\beta _{1l}$	0.60	0.001	0.001	95.2	$-$0.004	0.001	95.3
$\phi _l$	$-$1.30	$-$0.002	0.003	95.6	$-$0.001	0.003	95.3
$\gamma$	$-$1.50	$-$0.006	0.001	93.5	$-$0.005	0.001	92.5
(B) Recurrent events							
$\beta _{r}$	0.20	$-$0.010	0.002	94.6	$-$0.010	0.003	93.9
$\phi _1$	1.04	0.017	0.012	94.0	$-$0.025	0.014	94.1
$\phi _2$	0.93	0.027	0.023	95.4	0.032	0.050	95.2
$\phi _3$	0.79	0.032	0.040	93.8	0.046	0.072	95.6
$\alpha _1$	0.80	$-$0.037	0.018	93.6	$-$0.043	0.047	93.6
$\alpha _2$	0.53	$-$0.027	0.052	93.0	$-$0.059	0.079	94.9
$\eta _{r0}$	0.60	$-$0.012	0.004	93.6	$-$0.011	0.007	94.6
$\eta _{r1}$	0.60	$-$0.008	0.010	95.0	0.009	0.014	94.9
(C) Terminal event							
$\beta _{t}$	0.50	$-$0.029	0.005	90.9	$-$0.036	0.006	93.9
$\phi _{t}$	0.45	$-$0.025	0.017	95.0	$-$0.026	0.020	94.5
$\eta _{t0}$	0.90	$-$0.046	0.010	91.6	$-$0.049	0.014	94.3
$\eta _{t1}$	0.90	$-$0.045	0.027	93.2	0.048	0.035	93.0
$\zeta$	1.20	$-$0.080	0.029	87.1	$-$0.083	0.050	86.8
Variance/correlation							
$\sigma ^2_{b_0}$	1.25	$-$0.003	4.8$\times 10^{-4}$	94.6	$-$0.003	5.3$\times 10^{-4}$	94.8
$\sigma ^2_{b_1}$	0.80	$-$0.016	0.001	90.9	$-$0.014	0.001	90.8
$\rho _{01}$	0.50	0.035	0.002	93.9	0.035	0.002	94.2
$\sigma ^2_{\varepsilon }$	1.32	0.001	1.8$\times 10^{-5}$	95.3	0.001	2.2$\times 10^{-5}$	95.2
$\sigma ^2_{\nu }$	1.44	0.009	0.005	95.2	0.023	0.012	94.1

Given are bias, mean squared error (MSE), and average coverage probabilities (CP) of the 95% credible interval averaged over 300 datasets

**Table 7:** Simulation results for $n=4000$ and two censoring rates: 50% and 65%

		50% censoring			65% censoring		
		45% recurrent events			30% recurrent events		
	True	Bias	MSE	CP	Bias	MSE	CP
(A) Longitudinal							
$\beta _{0l}$	47.00	$-$0.001	0.002	95.4	$-$0.001	0.002	95.3
$\beta _{1l}$	0.60	$-$0.001	3.7 $\times 10^{-4}$	95.4	0.001	3.3 $\times 10^{-4}$	95.9
$\phi _l$	$-$1.30	$-$0.001	0.002	95.7	0.001	0.001	95.5
$\gamma$	$-$1.50	$-$0.001	0.001	95.8	$-$0.002	0.001	95.7
(B) Recurrent events							
$\beta _{r}$	0.20	$-$0.008	0.001	95.4	$-$0.005	0.001	95.3
$\phi _1$	1.04	0.002	0.005	95.7	0.004	0.007	95.5
$\phi _2$	0.93	0.010	0.012	95.6	0.011	0.020	95.4
$\phi _3$	0.79	0.015	0.021	96.4	0.028	0.044	96.8
$\alpha _1$	0.80	$-$0.025	0.010	94.4	$-$0.027	0.019	95.3
$\alpha _2$	0.53	$-$0.012	0.024	95.3	0.038	0.050	95.7
$\eta _{r0}$	0.60	$-$0.010	0.002	94.5	$-$0.009	0.004	95.3
$\eta _{r1}$	0.60	0.003	0.005	95.8	0.008	0.007	95.1
(C) Terminal event							
$\beta _{t}$	0.50	$-$0.016	0.003	94.1	$-$0.023	0.003	96.7
$\phi _{t}$	0.45	$-$0.006	0.006	96.0	$-$0.013	0.010	95.0
$\eta _{t0}$	0.90	$-$0.028	0.006	93.7	$-$0.030	0.007	95.4
$\eta _{t1}$	0.90	$-$0.018	0.011	95.0	$-$0.024	0.015	95.8
$\zeta$	1.20	$-$0.046	0.015	94.5	$-$0.048	0.028	93.3
Variance/correlation							
$\sigma ^2_{b_0}$	1.24	$-$0.002	2.3 $\times 10^{-4}$	95.2	$-$0.002	2.5 $\times 10^{-4}$	95.1
$\sigma ^2_{b_1}$	0.80	$-$0.010	0.001	93.5	$-$0.010	0.001	93.6
$\rho _{01}$	0.50	0.025	0.001	95.3	0.028	0.001	94.9
$\sigma ^2_{\varepsilon }$	1.32	0.001	1.2$\times 10^{-5}$	96.4	0.001	1.0 $\times 10^{-5}$	96.3
$\sigma ^2_{\nu }$	1.44	$-$0.006	0.003	95.7	0.016	0.005	95.5

Given are bias, mean squared error (MSE), and average coverage probabilities (CP) of the 95% credible interval averaged over 300 datasets

In the second part of the simulation study, we examined simpler analysis models that ignore the trivariate joint outcomes structure. Specifically, we examined (1) fitting models to each outcome individually (ignoring trivariate outcomes structure) and (2) fitting joint model of longitudinal and survival (time to terminal event), ignoring the effect of recurrent events process on the terminal event (relevant to the CRIC study; see Fig. 1). Results are presented in details in the Supplementary Materials. We briefly summarize here that estimation bias for the simpler models, individual outcome models as well as joint longitudinal and survival model, are several orders of magnitude larger relative to the trivariate model. Inference via credible intervals are also ineffective for the simpler models.

### Additional Simulation Studies and Results

We conducted additional simulation studies to examine and compare the efficacy of estimation and inference procedure. Specifically, these additional simulation studies examine the impact of (1) the variance of the frailty terms ($\sigma _{b_0}^2, \sigma _{b_1}^2, \sigma _\nu ^2$), (2) the correlation $\rho _{01}$, and (3) different baseline hazards (log-normal and Gompertz baseline hazards). For the variance of the frailty terms, we considered a “lower” ($\sigma _{b_0}^2=1.0, \sigma _{b_1}^2=0.5, \sigma _\nu ^2=1.2$) and a “higher” variance ($\sigma _{b_0}^2=2.0, \sigma _{b_1}^2=1.5, \sigma _\nu ^2=2.0$) setting relative to the main (mid-level) variance setting reported above in Sect. 4.1. We briefly summarize the results of these additional simulation studies:

- Variance of frailty terms: Supplementary Table S10A-B summarizes the results for the (A) lower and (B) higher frailty variance settings. The conclusions drawn from the results of these additional variance settings remain the same as previously reported above for the main simulation setting of Sect. 4.2. Overall, for most parameters (e.g., in the recurrent and terminal events), estimation bias was only slightly lower and higher corresponding the higher and lower variance settings relative to the mid-level/main setting. (e.g., compare to Table 7). The average coverage probabilities (CP) also target the 95% level in all settings.
- Correlation: In the main simulation study reported earlier, a correlation of 0.5 was used. We added a lower correlation setting of 0.3 for comparison. The performance of our estimation procedure in terms of bias, MSE, and confidence intervals under this setting is similar to the main simulation study. The results for the lower correlation setting is summarized in Supplementary Table S10C. Overall, for the 0.3 correlation setting, the average coverage probabilities (CP) target 95% as in the main simulation study with correlation of 0.5. Also, overall for most parameters, the biases were only slightly higher when the correlation is lower at 0.3.
- Baseline hazards: Similar to the reported results in the main simulation studies based on Weibull baseline hazard, the effectiveness of model estimation and inference similarly holds under log-normal and Gompertz baseline hazards. That is, low estimation bias observed for these additional baseline hazards were similar to the Weibull baseline hazard case and the coverage probabilities similarly target 95%. This is expected since the use of Bayesian P-splines to model the baseline hazard is flexibly adaptive to the form of the baseline hazard. Details of the simulation studies and results are provided in the Supplementary Materials and Supplementary Table S11.

## Discussion

Our work was motivated by the overall goal of CRIC study to better understand risk factors for three key interdependent outcomes processes, namely kidney disease progression, occurrence/recurrence of cardiovascular events, and eventual terminal event (kidney failure or death) in patients with mild to moderate CKD. The contribution of this work is primarily two-fold. First, this work fills an important methodology gap by developing a flexible Bayesian framework for trivariate joint modeling more generally. Although the work was motivated by and illustrated with an application with three outcome processes, the framework is flexible to accommodate more complex processes, such as multiple longitudinal markers and additional time-to-event processes.

The second contribution is that we formally addressed, for the first time in the literature to our knowledge, longstanding hypotheses regarding relationships among the *three key outcomes jointly* in patients with CKD. This novel analysis using the proposed trivariate joint model showed that eGFR decline is very strongly associated with kidney failure/death, as expected, but also that eGFR decline’s significant association with CV events was about 37% relative to the association with the terminal event. Also, a strong positive association was observed between recurrent CV events and the terminal event. Thus, the trivariate joint modeling allowed direct quantification of the relative strength of associations among the three key outcome processes.

Another new question we addressed is whether the effects of traditional risk factors, specifically diabetes, hypertension and (history of) CVD are event-varying in patients with CKD. We found that for patients with a history of CVD, the estimated risk of the first CV event is more than 2.5-fold higher compared to those without a history of CVD; however, the risk of the second and third CV events is reduced by more than half (although still significantly higher). This knowledge, combined with the strong links among the three outcomes, supports potential vigilant treatment/monitoring for mild/moderate CKD patients with a history of CVD to ameliorate the occurrence of the first CV event and recurrence of CV events. Additionally, we found that hypertension was associated with about 70% higher risk of having a first CV event, but was not associated with further events. Our analysis suggests/implies that treatment to control hypertension, not only serves to slow CKD progression, but also directly reduces the risk of an initial CV event. Finally, for diabetes, although there was no significant association with CV events directly, diabetes disease control through reduction of hemoglobin A1c was key to CV risk reduction.

Simulation studies conducted showed the effectiveness of estimation and inference for the proposed trivariate model. In practice, often simpler analysis models may be explored although we caution that simpler models that ignore the interdependency of the joint outcomes may lead to severely biased estimation and incorrect inference generally. This was illustrated in one of our simulation studies (details available in the online supplementary materials). R codes and documentation for fitting the proposed Bayesian trivariate joint model are made publicly available in Github to facilitate applications and further development.

Finally, we conclude with three notes regarding several areas of methodological and practical extensions of the current work. First, the proposed method can be extended to include cause-specific deaths (along with ESKD) under competing risks. Second, there is also an interest in developing a dynamic prediction framework in the context of joint modeling generally. More specifically, for the trivariate model proposed in this work, this extension requires nontrivial developments since the dynamic prediction would have to rely on the combined history of the longitudinal measurements and recurrent cardiovascular (CV) events. Under our proposed Bayesian framework, the dynamic prediction procedure would have to rely on sampling from the posterior distribution of the estimated parameter vector in addition to the posterior distribution of the random effects. However, the posterior distribution of the random effects would no longer follow a normal distribution conditional on the recurrent event history. Hence, sampling from the posterior distribution of the random effects would require the development of a Metropolis-Hastings algorithm with an appropriate proposal distribution that needs to be formulated. Although this methodological challenge is beyond the scope of the current applied work, its is an important direction for future research.

Third, the proposed methodology can be extended to complex joint models with other association structures (e.g., see [40, 41]) in addition to the shared random effects approach considered in this work. More general association structures between the longitudinal and recurrent and between longitudinal and terminal event processes, similar to the proposed association framework in Król et al. [28] may be of interest. Because in our trivariate model, the three submodels do not share the same time index, it is not straight forward to incorporate other forms of the longitudinal history directly in the recurrent and survival submodels.

## Supplementary Information

Below is the link to the electronic supplementary material.

Supplementary file 1 (pdf 788 KB)

## Data Availability

The release of the data used in this paper is governed by NIDDK through a data use agreement (https://repository.niddk.nih.gov/studies/cric/).

## Declarations

### Supplementary Information and R Code

The supplementary material for this article is available online. The R code and documentation for implementing the proposed Bayesian trivariate joint model are provided on Github at https://github.com/esrakurum/Bayes-Trivariate.
