# Supplementary material for: A Bayesian Joint Model of Longitudinal Kidney Disease Progression, Recurrent Cardiovascular Events, and Terminal Event in Patients with Chronic Kidney Disease
Source: Stat Biosci. 2024 May 9;17(2):528–54. doi: 10.1007/s12561-024-09429-6 (PMC12122633; doi:10.1007/s12561-024-09429-6)
Supplement: Supplementary file 1 — (pdf 788 KB) [file 12561_2024_9429_MOESM1_ESM.pdf]

Supplementary Materials

A Bayesian joint model of longitudinal kidney disease progression, recurrent cardiovascular events, and terminal event in patients with chronic kidney disease

Esra Kürüm[1], Brian Kwan[2*], Qi Qian[2], Sudipto Banerjee[2], Connie M. Rhee[4,5], Danh V. Nguyen[3†], and Damla Şentürk[2]

[1]Department of Statistics, University of California, Riverside, CA 92521, USA
[2]Department of Biostatistics, University of California, Los Angeles, CA 90095, USA
[3]Department of Medicine, University of California Irvine, Orange, CA 92868, USA
[4]Department of Medicine, University of California, Los Angeles, CA 90095, USA
[5]Nephrology Section, VA Greater Los Angeles Health Care System, Los Angeles, CA 90073, USA

*co-first author
†Correspondence: Danh V. Nguyen, University of California Irvine, Orange, CA 92868, USA. Email: danhvn1@hs.uci.edu

1 Simulation studies: Simpler models

Additional simulation studies examined the impact of using simpler models that do not take full advantage of the interdependent trivariate outcomes. More specifically, we examined simpler analysis models that ignore the trivariate joint outcomes structure by (a) fitting an individual model to each outcome separately (which ignores the trivariate outcomes structure) and (b) fitting a joint model of longitudinal and terminal event (which ignores the effect of recurrent events process on the terminal event; see Figure 1 in the main paper). Results are summarized in Table S1-S2 for the three individual models in (b) and in Table S3-S4 for the joint model of longitudinal and terminal event in (b).

We examined the relative bias in estimation of the simpler modeld relative to the full trivariate model. With respect to estimation (e.g., at $n = 2000$), the individual models have biases that were up to several folds higher compared to the full trivariate model for the longitudinal outcome (relative bias ranging from 33% to 1050%), recurrent events outcome (relative bias ranging from 200% to 470%), and terminal event outcome (relative bias ranging from 227% to 272%). For the simpler joint model of longitudinal and terminal event, biases in parameter estimates range from 33% to 300% for the longitudinal submodel and 126% to 248% for the terminal event submodel. Similar levels of bias were found for the $n = 4000$ setting.

With respect to inference, simpler models (a) and (b) were found to inadequate. Figure S1 summarizes the coverage probabilities (CP) of 95% credible intervals (dashed line at 95%). The CP for the trivariate model targets the 95% level and improves with increasing sample size ($n = 2000$, 4000) (Figure S1(a)). For the simpler models (individual models fitted to each outcome separately and simpler joint model of longitudinal and terminal event outcomes) CP are substantially below the 95% target (Figure S1(b)-(c)). Thus, such simpler models in the context of interdependent trivariate outcomes generally can result in misleading inference.

2 Trace plots and MCMC convergence

To assess satisfactory MCMC samples for the trivariate model presented in Section 3, trace plots for model parameters are provided in Figure S2, S3, and S4 for the longitudinal, recurrent events, and terminal event submodels, respectively. The results show satisfactory MCMC convergence for all model parameters. We also examined the scale reduction factor, $R \approx 1$, as suggested by Gelman and Rubin (1992) to monitor convergence.

3 Sensitivity analyses: Hyperparameters

Analyses of the CRIC data were conducted to assess sensitivity to hyperparameters. Table S5, S6, and S7 summarizes sensitivity analyses that uses inverse gamma IG(1, 0.05) and IG (1, 0.5) compared to the main results reported in the paper using IG(1, 0.005) for the longitudinal, recurrent, and terminal event model components, respectively. These sensitivity analyses show that main results reported are fairly robust to hyperparameter choice. Additionally, Table S8 summarizes the sensitivity analyses with respect to the estimation and inference for the model association parameters and variance components (and correlation). Similar the estimation and inference for the covariate effects, the linkage and variance components parameters were not sensitive to hyperparameter choice.

4 Simple analyses of outcomes separately in the CRIC data

Simple analysis models examining the effects of covariates on each outcome separately (longitudinal eGFR, recurrent CV events, and terminal event) are summarized in Table S9. As described in more details in the main paper these simple analyses which ignore the interdependent outcomes can result in biased estimates and invalid inference. Thus, they should be interpreted with caution in light of potentially biased estimation and invalid inference.

5 Additional simulation studies

Additional simulation with respect the variance of the frailty terms, correlation, and different baseline hazards were conducted. More specifically, the following simulations settings were examined:

- **Variance of frailty terms:** Two simulation settings with higher ($\sigma_{b0}^2 = 2.00$, $\sigma_{b1}^2 = 1.50$, and $\sigma_{\nu}^2 = 2.00$) and lower ($\sigma_{b0}^2 = 1.00$, $\sigma_{b1}^2 = 0.50$, and $\sigma_{\nu}^2 = 1.20$) variance of the frailty terms settings. The results are summarized in Table S10.

- **Correlation:** Additional simulation with a lower correlation (ρ_b) of 0.3 is summarized in Table S10.

- **Baseline hazards:** Additional simulation studies with log-normal ($\mu = 1.75$ and $\sigma = 0.5$ for the terminal event, and $\mu = 1.5$ and $\sigma = 0.5$ for the recurrent event) and Gompertz (shape parameter $\alpha = 1.1$ and scale parameter $\lambda = 0.01$ for the terminal event, and $\alpha = 2.1$ and $\lambda = 0.01$ for the recurrent event) baseline hazard functions are summarized in Table S11.

6 Bayesian model fit: Deviance information criterion (DIC)

To assess a Bayesian model fit that penalizes for model complexity, deviance information criterion (DIC) can be conveniently computed based on samples generated by the MCMC simulation (Spiegelhalter et al. 2002; Gelman et al. 2014). DICs were computed for the the the following simpler models: null model (excluding all covariates) and three "bivariate" models that includes only two distinct linkages among the three outcomes.

Simpler models:

(M0) Null model:

$$\begin{aligned} Y_i(t) &= \beta_0 + b_{i0} + \varepsilon_i(t), \\ r_{ij}(t \mid \mathbf{b}_i, \nu_i) &= h_{r0}(t) \exp\left(\eta_{r0} b_{i0} + \nu_i\right), \\ h_i(t \mid \mathbf{b}_i, \nu_i) &= h_{t0}(t) \exp(\eta_{t0} b_{i0} + \zeta \nu_i). \end{aligned}$$

(M1) Bivariate 1 – longitudinal linked with survival and recurrent, but survival and recurrent are not linked:

$$\begin{aligned} Y_i(t) &= \beta_0 + \mathbf{X}_i^{\mathrm{T}} \boldsymbol{\beta}_l + \mathbf{Z}_i^{\mathrm{T}} \boldsymbol{\phi}_l + \gamma t + b_{i0} + b_{i1} t + \varepsilon_i(t), \\ r_{ij}(t \mid \mathbf{X}_i, \mathbf{Z}_i, \mathbf{b}_i, \nu_i) &= h_{r0}(t) \exp\left(\mathbf{X}_i^{\mathrm{T}} \boldsymbol{\beta}_r + \mathbf{Z}_i^{\mathrm{T}} \boldsymbol{\phi}_{rj} + \sum_{m=0}^{j-1} \alpha_m + \eta_{r0} b_{i0} + \eta_{r1} b_{i1} + \nu_i\right), \\ h_i(t \mid \mathbf{X}_i, \mathbf{Z}_i, \mathbf{b}_i, \nu_i) &= h_{t0}(t) \exp(\mathbf{X}_i^{\mathrm{T}} \boldsymbol{\beta}_t + \mathbf{Z}_i^{\mathrm{T}} \boldsymbol{\phi}_t + \eta_{t0} b_{i0} + \eta_{t1} b_{i1} \cancel{+ \zeta \nu_i}). \end{aligned}$$

(M2) Bivariate 2 – survival linked with longitudinal and recurrent, but longitudinal and recurrent are not linked:

$$\begin{aligned} Y_i(t) &= \beta_0 + \mathbf{X}_i^{\mathrm{T}} \boldsymbol{\beta}_l + \mathbf{Z}_i^{\mathrm{T}} \boldsymbol{\phi}_l + \gamma t + b_{i0} + b_{i1} t + \varepsilon_i(t), \\ r_{ij}(t \mid \mathbf{X}_i, \mathbf{Z}_i, \mathbf{b}_i, \nu_i) &= h_{r0}(t) \exp\left(\mathbf{X}_i^{\mathrm{T}} \boldsymbol{\beta}_r + \mathbf{Z}_i^{\mathrm{T}} \boldsymbol{\phi}_{rj} + \sum_{m=0}^{j-1} \alpha_m + \cancel{\eta_{r0} b_{i0} + \eta_{r1} b_{i1}} + \nu_i\right), \\ h_i(t \mid \mathbf{X}_i, \mathbf{Z}_i, \mathbf{b}_i, \nu_i) &= h_{t0}(t) \exp(\mathbf{X}_i^{\mathrm{T}} \boldsymbol{\beta}_t + \mathbf{Z}_i^{\mathrm{T}} \boldsymbol{\phi}_t + \eta_{t0} b_{i0} + \eta_{t1} b_{i1} + \zeta \nu_i). \end{aligned}$$

(M3) Bivariate 3 – recurrent linked with longitudinal and survival, but longitudinal and survival are not linked:

$$\begin{aligned} Y_i(t) &= \beta_0 + \mathbf{X}_i^{\mathrm{T}} \boldsymbol{\beta}_l + \mathbf{Z}_i^{\mathrm{T}} \boldsymbol{\phi}_l + \gamma t + b_{i0} + b_{i1} t + \varepsilon_i(t), \\ r_{ij}(t \mid \mathbf{X}_i, \mathbf{Z}_i, \mathbf{b}_i, \nu_i) &= h_{r0}(t) \exp\left(\mathbf{X}_i^{\mathrm{T}} \boldsymbol{\beta}_r + \mathbf{Z}_i^{\mathrm{T}} \boldsymbol{\phi}_{rj} + \sum_{m=0}^{j-1} \alpha_m + \eta_{r0} b_{i0} + \eta_{r1} b_{i1} + \nu_i\right), \\ h_i(t \mid \mathbf{X}_i, \mathbf{Z}_i, \mathbf{b}_i, \nu_i) &= h_{t0}(t) \exp(\mathbf{X}_i^{\mathrm{T}} \boldsymbol{\beta}_t + \mathbf{Z}_i^{\mathrm{T}} \boldsymbol{\phi}_t + \cancel{\eta_{t0} b_{i0} + \eta_{t1} b_{i1}} + \zeta \nu_i). \end{aligned}$$

7 Implementation and R codes

All computations were performed in R (version 4.0.2), and as there are no closed-form solutions for the posterior distributions, we fitted our model using the Bayesian software JAGS (version 4.3.0) via the `rjags` package (Plummer et al. 2019). JAGS uses a combination of Metropolis sampling, Gibbs sampling, and other MCMC algorithms while fitting the models (Plummer 2017). We note that the sampling methods are chosen automatically for all parameters during the initialization of a model in the implementation in JAGS. More specifically, JAGS holds a list of sampler objects, which inspect the graph (Bayesian graphical model), recognize sets of parameters that can be updated with specific methods, and generate sampler objects for the parameters starting with the most efficient sampling method. The user has some control over the implementation of the sampling procedure via loading modules. We took advantage of this capability and before running our JAGS code, we loaded the 'glm' module to ensure we use samplers built for efficient updating of generalized linear mixed models. (This module is mainly built on top of the Csparse and CHOLMOD sparse matrix libraries (Davis 2006; Davis and Hager 1999), which allows for updating both fixed and random effects in the same block.) R codes and documentation for fitting the proposed trivariate joint model are made publicly available at https://github.com/esrakurum/Bayes-Trivariate.

References

[1] Davis TA (2006) Direct methods for sparse linear systems. Society for Industrial and Applied Mathematics, Philadelphia, PA.

[2] Davis TA, Hager WW (1999) Modifying a sparse Cholesky factorization. SIAM Journal on Matrix Analysis and Applications 20(3): 606-627. https://doi.org/10.1137/S0895479897321076

[3] Gelman A, Rubin DB (1992) Inference from iterative simulation using multiple sequences. Statistical Science 7(4): 457–472. https://doi.org/10.1214/ss/1177011136

[4] Gelman A, Carlin JB, Stern HS, Dunson DB, Vehtari A, Rubin DB (2014). Bayesian Data Analysis. Chapman and Hall/CRC, Boca Raton, FL.

[5] Plummer M (2017) JAGS version 4.3. 0 user manual [computer software manual]. sourceforge net/projects/mcmc-jags/files. Manuals/4 x, 2.

[6] Plummer M, Stukalov A, Denwood M. rjags: Bayesian graphical models using MCMC. R package version, 4(10), 2019.

[7] Spiegelhalter DJ, Best NG, Carlin BP and Van der Linde A (2002) Bayesian measures of model complexity and fit (with discussion). Journal of the Royal Statistical Society, Series B, 64(4):583–616. https://doi.org/10.1111/1467-9868.00353

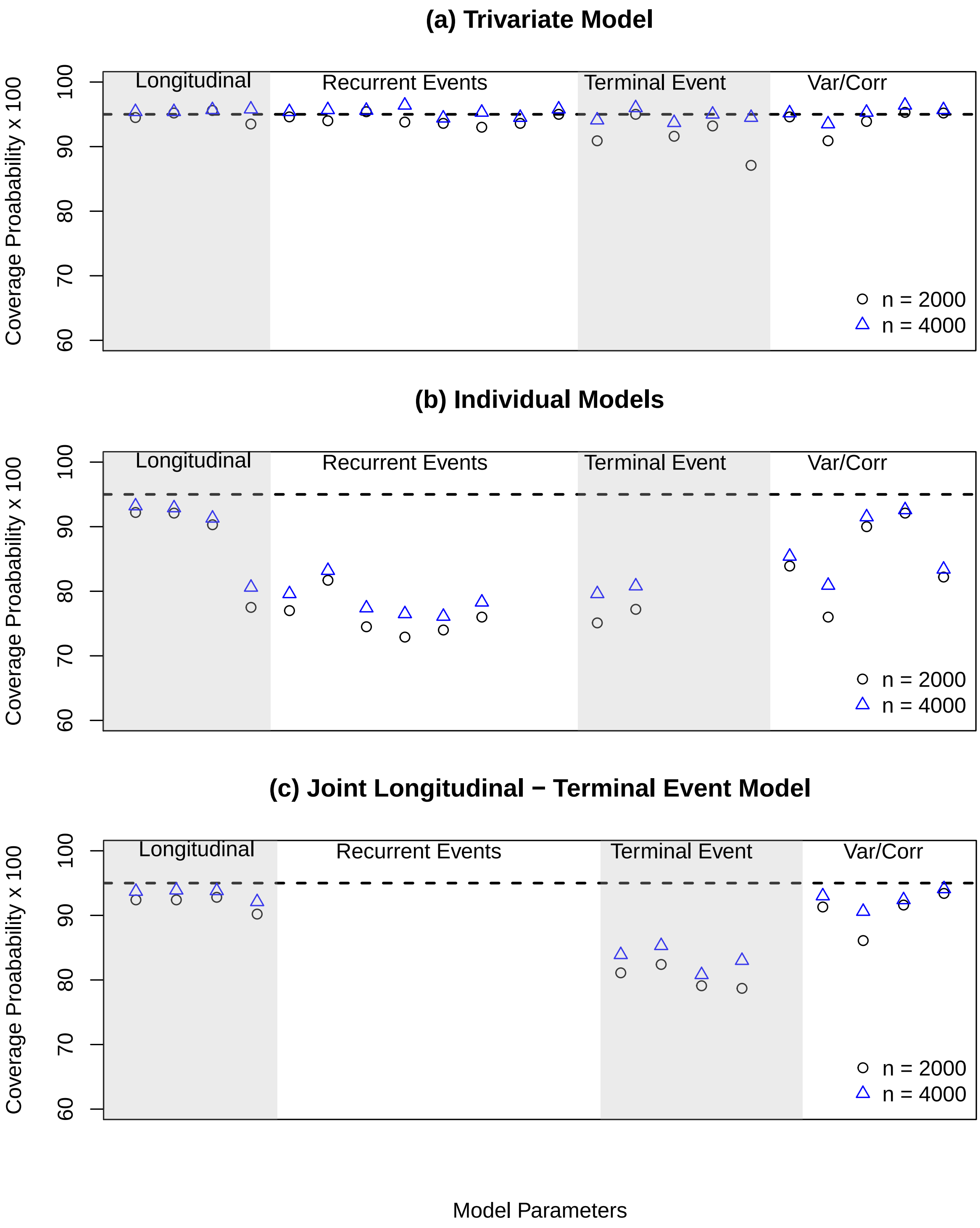

Figure S1: Ineffectiveness of simpler models, ignoring trivariate outcomes structure, for inference. (a) Coverage probabilities (CP) of 95% credible intervals (dashed line at 95%) for the full trivariate model correctly targets 95%. See also main text Table 4 and 5. (b) Simpler individual models fitted to each outcome separately have poor CP below 95%. See also Table S1 and S2. (c) Simpler joint model of longitudinal and terminal event outcomes have poor CP below 95%, especially for terminal event parameters, variance components, and correlation parameter. See also Table S3 and S4. (Horizontal axis are model parameters as listed in Table 1.)

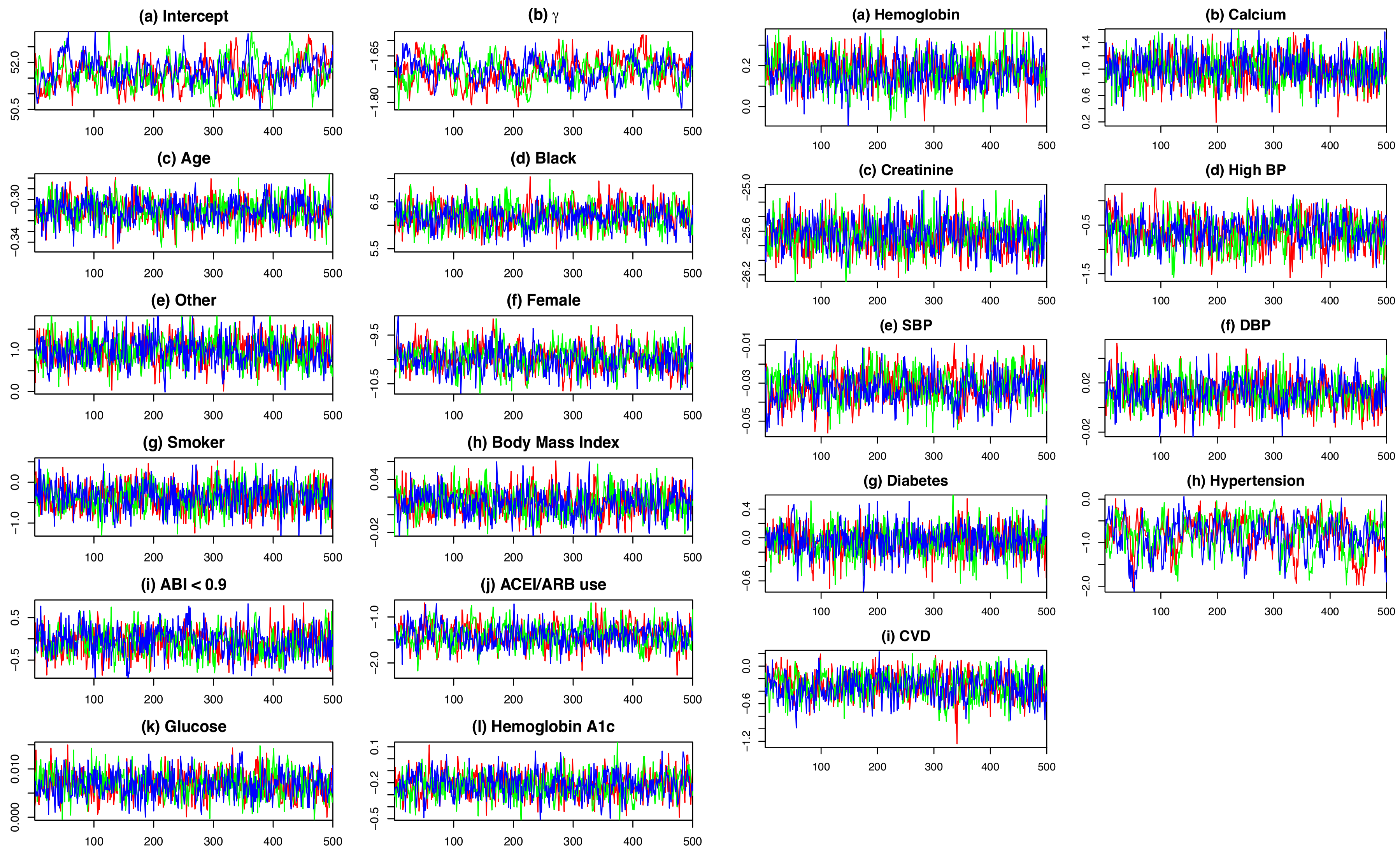

Figure S2: Trace plots for longitudinal submodel parameters.

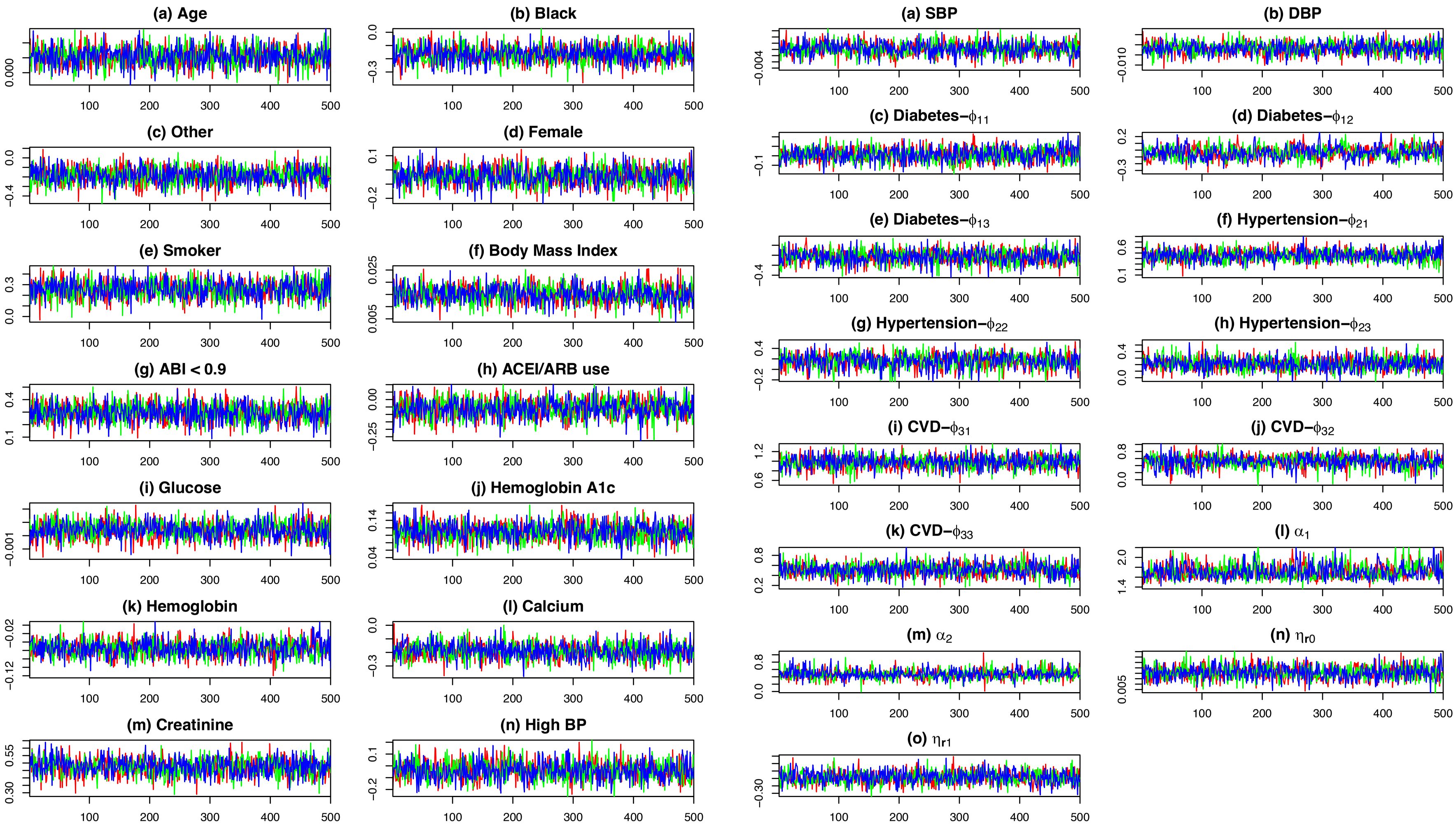

Figure S3: Trace plots for recurrent events submodel parameters.

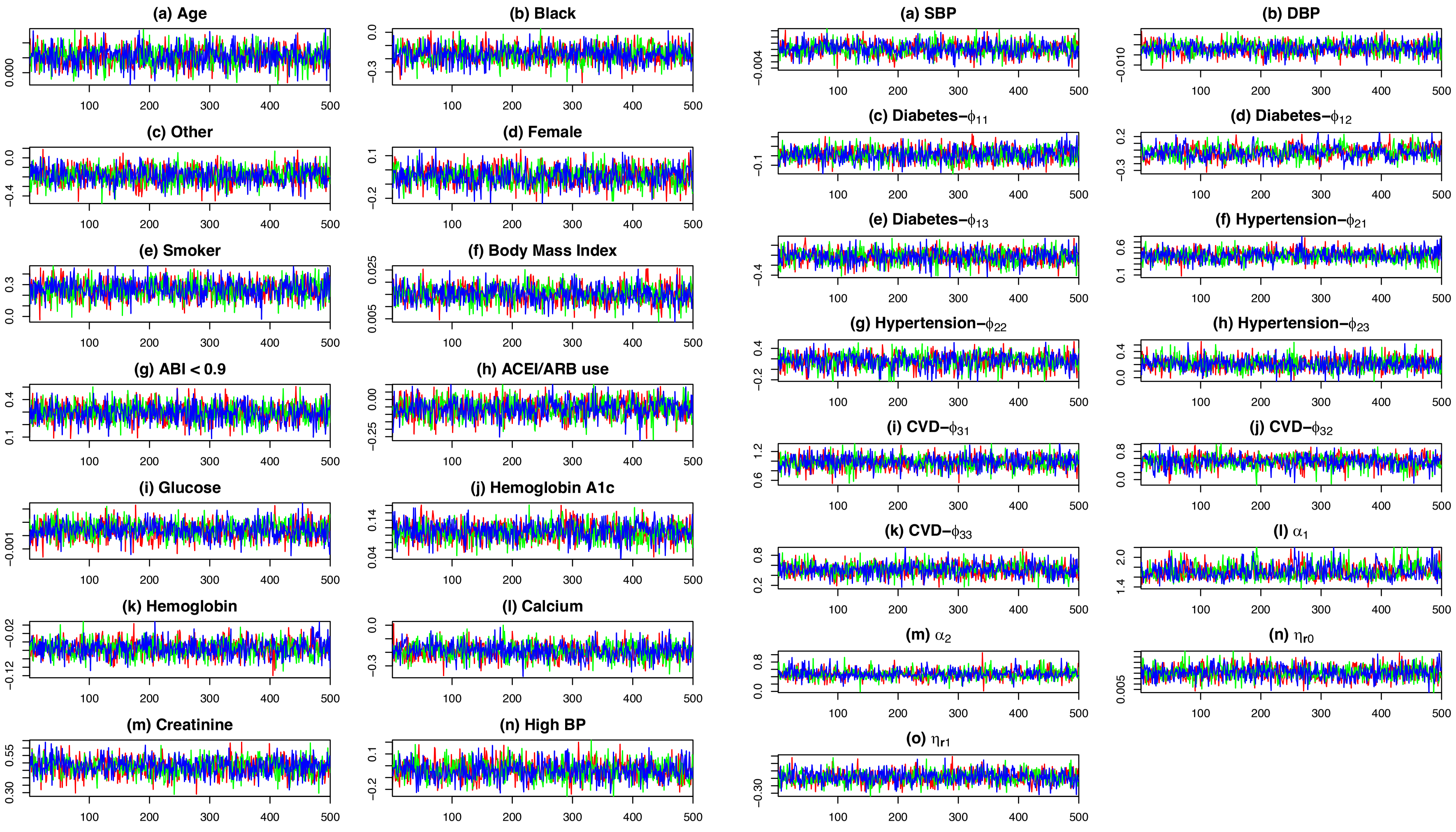

Figure S4: Trace plots for terminal event submodel parameters.

Table S1: Simulation results for simpler individual models for each outcome separately with $n = 2000$ and two censoring rates: 50% and 65%. Given are bias, mean squared error (MSE), and average coverage probabilities (CP) of the 95% credible interval averaged over 300 datasets.

		50% Censoring 45% Recurrent Events			65% Censoring 30% Recurrent Events		
	True	Bias	MSE	CP	Bias	MSE	CP
Longitudinal							
β_{0l}	47.00	0.004	0.003	92.2	-0.011	0.005	91.7
β_{1l}	0.60	0.004	0.002	92.1	-0.007	0.002	92.4
ϕ_l	-1.30	-0.010	0.003	90.3	0.012	0.003	90.1
γ	-1.50	-0.069	0.006	77.5	-0.064	0.006	77.8
Recurrent Events							
β_r	0.20	-0.057	0.016	77.0	-0.059	0.014	77.4
ϕ_1	1.04	0.085	0.020	81.7	0.094	0.023	81.0
ϕ_2	0.93	0.096	0.045	74.5	0.098	0.063	72.7
ϕ_3	0.79	0.110	0.052	72.9	0.129	0.091	73.0
α_1	0.80	-0.118	0.060	74.0	-0.144	0.082	75.1
α_2	0.53	-0.081	0.068	76.0	-0.113	0.089	76.9
Terminal Event							
β_t	0.50	-0.095	0.057	75.1	-0.110	0.060	75.3
ϕ_t	0.45	-0.093	0.057	77.2	-0.108	0.061	77.6
Variance/Correlation							
$\sigma^2_{b_0}$	1.25	-0.010	0.002	83.9	-0.011	0.001	84.0
$\sigma^2_{b_1}$	0.80	-0.052	0.004	76.0	-0.047	0.003	79.7
ρ_{01}	0.50	0.045	0.003	90.0	0.041	0.002	90.1
σ^2_{ε}	1.32	0.003	3.1×10^{-5}	92.1	0.003	3.2×10^{-5}	92.2
σ^2_{ν}	1.44	0.062	0.031	82.2	0.073	0.043	81.0

Table S2: Simulation results for simpler individual models for each outcome separately with $n = 4000$ and two censoring rates: 50% and 65%. Given are bias, mean squared error (MSE), and average coverage probabilities (CP) of the 95% credible interval averaged over 300 datasets.

		50% Censoring 45% Recurrent Events			65% Censoring 30% Recurrent Events		
	True	Bias	MSE	CP	Bias	MSE	CP
Longitudinal							
β_{0l}	47.00	0.003	0.002	93.2	0.006	0.003	92.8
β_{1l}	0.60	0.003	0.002	92.9	-0.004	0.002	93.1
ϕ_l	-1.30	-0.007	0.002	91.3	0.010	0.002	91.0
γ	-1.50	-0.060	0.005	80.6	-0.061	0.005	81.7
Recurrent Events							
β_r	0.20	-0.049	0.015	79.6	-0.046	0.012	79.4
ϕ_1	1.04	0.044	0.016	83.2	0.050	0.014	83.3
ϕ_2	0.93	0.056	0.021	77.4	0.060	0.038	77.1
ϕ_3	0.79	0.068	0.032	76.5	0.081	0.057	77.0
α_1	0.80	-0.051	0.034	76.1	-0.073	0.045	76.4
α_2	0.53	-0.069	0.036	78.3	-0.078	0.052	78.1
Terminal Event							
β_t	0.50	-0.057	0.030	79.6	-0.068	0.033	80.1
ϕ_t	0.45	-0.043	0.036	80.8	-0.055	0.041	80.4
Variance/Correlation							
$\sigma^2_{b_0}$	1.25	-0.008	0.001	85.4	-0.009	0.001	85.3
$\sigma^2_{b_1}$	0.80	-0.041	0.003	80.9	-0.039	0.003	80.6
ρ_{01}	0.50	0.034	0.002	91.5	0.035	0.002	91.3
σ^2_{ε}	1.32	0.002	2.4×10^{-5}	92.6	0.002	2.7×10^{-5}	92.8
σ^2_{ν}	1.44	0.045	0.028	83.4	0.050	0.029	83.0

Table S3: Simulation results for a simpler joint model for longitudinal and terminal event at $n = 2000$ and two censoring rates: 50% and 65%. Given are bias, mean squared error (MSE), and average coverage probabilities (CP) of the 95% credible interval averaged over 300 datasets.

		50% Censoring 45% Recurrent Events			65% Censoring 30% Recurrent Events		
	True	Bias	MSE	CP	Bias	MSE	CP
Longitudinal							
β_{0l}	47.00	-0.004	0.003	92.4	-0.010	0.005	92.6
β_{1l}	0.60	0.002	0.002	92.4	-0.005	0.002	92.8
ϕ_l	-1.30	-0.008	0.003	92.8	-0.010	0.003	92.6
γ	-1.50	-0.015	0.003	90.2	-0.016	0.003	90.5
Terminal Event							
β_t	0.50	-0.081	0.038	81.1	-0.084	0.040	81.3
ϕ_t	0.45	-0.087	0.041	82.4	-0.085	0.044	83.3
η_{t0}	0.90	-0.104	0.042	79.1	-0.105	0.057	79.2
η_{t1}	0.90	-0.121	0.069	78.7	-0.126	0.071	78.0
ζ	1.20						
Variance/Correlation							
$\sigma^2_{b_0}$	1.25	-0.005	0.001	91.3	-0.005	0.001	90.2
$\sigma^2_{b_1}$	0.80	-0.027	0.002	86.1	-0.024	0.002	86.6
ρ_{01}	0.50	0.040	0.003	91.6	0.039	0.002	91.7
σ^2_{ε}	1.32	0.002	2.3×10^{-5}	93.4	0.002	2.5×10^{-5}	93.8

Table S4: Simulation results for a simpler joint model for longitudinal and terminal event at $n = 4000$ and two censoring rates: 50% and 65%. Given are bias, mean squared error (MSE), and average coverage probabilities (CP) of the 95% credible interval averaged over 300 datasets.

		50% Censoring 45% Recurrent Events			65% Censoring 30% Recurrent Events		
	True	Bias	MSE	CP	Bias	MSE	CP
Longitudinal							
β_{0l}	47.00	-0.003	0.002	93.7	-0.004	0.003	93.5
β_{1l}	0.60	0.001	0.002	93.9	-0.002	0.002	93.4
ϕ_l	-1.30	-0.005	0.002	93.8	-0.006	0.002	93.3
γ	-1.50	-0.010	0.002	92.1	-0.014	0.003	92.5
Terminal Event							
β_t	0.50	-0.052	0.022	83.9	-0.053	0.021	84.0
ϕ_t	0.45	-0.049	0.026	85.3	-0.050	0.028	85.2
η_{t0}	0.90	-0.078	0.032	80.8	-0.082	0.031	81.0
η_{t1}	0.90	-0.068	0.051	83.0	-0.069	0.057	83.4
ζ	1.20						
Variance/Correlation							
$\sigma^2_{b_0}$	1.25	-0.004	0.001	93.0	-0.004	0.001	93.3
$\sigma^2_{b_1}$	0.80	-0.018	0.002	90.6	-0.019	0.002	90.5
ρ_{01}	0.50	0.030	0.002	92.4	0.031	0.002	92.1
σ^2_{ε}	1.32	0.001	2.0×10^{-5}	94.1	0.001	1.7×10^{-5}	94.6

Table S5: Longitudinal component. Sensitivity analyses for hyperparameter: (A) Main analysis with inverse gamma IG(1, 0.005); (B) sensitivity analysis with IG(1, 0.05); (C) sensitivity analysis with IG(1, 0.5).

**Variable**	**(A) IG(1, 0.005)** **Estimate (95% CI)**	**(B) IG(1, 0.05)** **Estimate (95% CI)**	**(C) IG(1, 0.5)** **Estimate (95% CI)**
Intercept	52.191 (51.452, 52.941)*	52.204 (51.444, 52.953)*	52.222 (51.444, 52.953)*
Time, γ	-1.723 (-1.793, -1.653)*	-1.721 (-1.810, -1.637)*	-1.736 (-1.811, -1.659)*
Age (years)	-0.306 (-0.327, -0.284)*	-0.305 (-0.328, -0.283)*	-0.305 (-0.328, -0.283)*
Black (ref. white)	6.242 (5.766, 6.740)*	6.243 (5.770, 6.730)*	6.249 (5.783, 6.712)*
Other	0.980 (0.368, 1.611)*	0.996 (0.369, 1.649)*	0.978 (0.348, 1.628)*
Female	-10.011 (-10.492, -9.528)*	-10.002 (-10.480, -9.527)*	-10.001 (-10.491, -9.535)*
Smoker	-0.354 (-0.983, 0.282)	-0.354 (-0.968, 0.272)	-0.341 (-0.932, 0.268)
Body Mass Index	0.016 (-0.013, 0.044)	0.017 (-0.012, 0.045)	0.017 (-0.010, 0.045)
ABI < 0.9	-0.022 (-0.608, 0.559)	-0.024 (-0.641, 0.560)	-0.027 (-0.615, 0.565)
ACEI/ARB use	-1.239 (-1.706, -0.802)*	-1.212 (-1.677, -0.746)*	-1.196 (-1.638, -0.732)*
Glucose (mg/dL)	0.007 (0.002, 0.012)*	0.007 (0.002, 0.012)*	0.007 (0.002, 0.012)*
Hemoglobin A1c (%)	-0.204 (-0.395, -0.003)*	-0.211 (-0.409, -0.015)*	-0.211 (-0.398, -0.012)*
Hemoglobin (g/dL)	0.163 (0.019, 0.301)*	0.164 (0.019, 0.309)*	0.159 (0.013, 0.299)*
Calcium (mg/dL)	1.010 (0.581, 1.414)*	1.017 (0.585, 1.446)*	1.003 (0.586, 1.423)*
Creatinine	-25.632 (-26.072, -25.214)*	-25.611 (-26.022, -25.190)*	-25.621 (-26.060, -25.193)*
High BP	-0.636 (-1.203, -0.027)*	-0.61 (-1.205, -0.003)*	-0.596 (-1.195, 0.001)
SBP (mmHg)	-0.03 (-0.045, -0.015)*	-0.03 (-0.046, -0.015)*	-0.03 (-0.046, -0.015)*
DPB (mmHg)	0.014 (-0.009, 0.037)	0.014 (-0.008, 0.038)	0.015 (-0.008, 0.038)
Diabetes	0.072 (-0.587, 0.415)	0.059 (-0.564, 0.434)	0.056 (-0.582, 0.473)
Diabetes - ϕ_{11}	-	-	-
Diabetes - ϕ_{12}	-	-	-
Diabetes - ϕ_{13}	-	-	-
Hypertension	-1.500 (-2.156, -0.864)*	-1.561 (-2.202, -0.919)*	-1.604 (-2.233, -1.004)*
Hypertension - ϕ_{21}	-	-	-
Hypertension - ϕ_{22}	-	-	-
Hypertension - ϕ_{23}	-	-	-
CVD	-0.483 (-0.908, -0.066)*	-0.485 (-0.926, -0.051)*	-0.502 (-0.932, -0.058)*
CVD - ϕ_{31}	-	-	-
CVD - ϕ_{32}	-	-	-
CVD - ϕ_{33}	-	-	-
α_1	-	-	-
α_2	-	-	-

*95% credible interval (CI) does not include estimate of 0;
angiotensin-converting enzyme inhibitor(ACEI); angiotensin receptor blocker (ARB); blood pressure (BP); cardiovascular disease (CVD); hemoglobin A1c (HbA1c); systolic and diastolic BP (SBP, DBP)

Table S6: Recurrent events component. Sensitivity analyses for hyperparameter: (A) Main analysis with inverse gamma IG(1, 0.005); (B) sensitivity analysis with IG(1, 0.05); (C) sensitivity analysis with IG(1, 0.5).

**Variable**	**(A) IG(1, 0.005)** **HR (95% CI)**	**(B) IG(1, 0.05)** **HR (95% CI)**	**(C) IG(1, 0.5)** **HR (95% CI)**
Intercept	-	-	-
Time, γ	-	-	-
Age (years)	1.034 (1.027, 1.040)*	1.034 (1.027, 1.040)*	1.034 (1.027, 1.039)*
Black (ref. white)	0.856 (0.764, 0.958)*	0.860 (0.770, 0.960)*	0.860 (0.766, 0.958)*
Other	0.814 (0.694, 0.953)*	0.818 (0.700, 0.955)*	0.820 (0.696, 0.951)*
Female	0.960 (0.858, 1.074)	0.964 (0.862, 1.078)	0.963 (0.856, 1.075)
Smoker	1.296 (1.119, 1.492)*	1.294 (1.124, 1.487)*	1.296 (1.120, 1.492)*
Body Mass Index	1.015 (1.009, 1.022)*	1.015 (1.009, 1.022)*	1.015 (1.009, 1.022)*
ABI < 0.9	1.346 (1.196, 1.511)*	1.344 (1.192, 1.517)*	1.342 (1.190, 1.511)*
ACEI/ARB use	0.944 (0.845, 1.055)	0.942 (0.843, 1.051)	0.943 (0.843, 1.057)
Glucose (mg/dL)	1.000 (0.999, 1.001)	1.000 (0.999, 1.001)	1.000 (0.999, 1.001)
Hemoglobin A1c (%)	1.115 (1.067, 1.163)*	1.114 (1.067, 1.162)*	1.114 (1.068, 1.161)*
Hemoglobin (g/dL)	0.961 (0.931, 0.993)*	0.962 (0.931, 0.993)*	0.962 (0.931, 0.992)*
Calcium (mg/dL)	0.824 (0.742, 0.908)*	0.822 (0.743, 0.907)*	0.822 (0.746, 0.909)*
Creatinine	1.603 (1.446, 1.784)*	1.605 (1.448, 1.782)*	1.605 (1.443, 1.775)*
High BP	0.969 (0.838, 1.117)	0.972 (0.840, 1.121)	0.972 (0.837, 1.124)
SBP (mmHg)	1.002 (0.999, 1.006)	1.002 (0.998, 1.006)	1.002 (0.998, 1.006)
DPB (mmHg)	0.998 (0.993, 1.003)	0.998 (0.993, 1.003)	0.998 (0.993, 1.003)
Diabetes	-	-	-
Diabetes - ϕ_{11}	1.151 (1.000, 1.330)	1.157 (1.000, 1.331)	1.157 (1.0002, 1.324)*
Diabetes - ϕ_{12}	0.969 (0.803, 1.172)	0.969 (0.795, 1.170)	0.969 (0.797, 1.164)
Diabetes - ϕ_{13}	0.808 (0.643, 1.026)	0.812 (0.643, 1.026)	0.812 (0.636, 1.025)
Hypertension	-	-	-
Hypertension - ϕ_{21}	1.586 (1.259, 2.004)*	1.611 (1.293, 2.000)*	1.611 (1.273, 1.970)*
Hypertension - ϕ_{22}	1.179 (0.868, 1.621)	1.170 (0.835, 1.631)	1.17 (0.839, 1.616)
Hypertension - ϕ_{23}	1.234 (0.800, 1.896)	1.255 (0.841, 1.906)	1.255 (0.812, 1.925)
CVD	-	-	-
CVD - ϕ_{31}	2.643 (2.342, 2.986)*	2.649 (2.351, 2.989)*	2.649 (2.342, 2.983)*
CVD - ϕ_{32}	1.718 (1.451, 2.040)*	1.721 (1.449, 2.034)*	1.721 (1.448, 2.026)*
CVD - ϕ_{33}	1.675 (1.346, 2.088)*	1.680 (1.355, 2.083)*	1.680 (1.342, 2.096)*
α_1	5.468 (3.755, 7.949)*	5.618 (3.792, 8.158)*	5.618 (3.881, 8.125)*
α_2	1.597 (0.938, 2.713)	1.554 (0.922, 2.524)	1.554 (0.929, 2.557)

*95% credible interval (CI) does not include hazard ratio (HR) of 1;
angiotensin-converting enzyme inhibitor(ACEI); angiotensin receptor blocker (ARB); blood pressure (BP); cardiovascular disease (CVD); hemoglobin A1c (HbA1c); systolic and diastolic BP (SBP, DBP)

Table S7: Terminal events component. Sensitivity analyses for hyperparameter: (A) Main analysis with inverse gamma IG(1, 0.005); (B) sensitivity analysis with IG(1, 0.05); (C) sensitivity analysis with IG(1, 0.5).

Variable	(A) IG(1, 0.005) HR (95% CI)	(B) IG(1, 0.05) HR (95% CI)	(C) IG(1, 0.5) HR (95% CI)
Intercept	-	-	
Time, γ	-	-	
Age (years)	1.028 (1.021, 1.036)*	1.028 (1.021, 1.036)*	1.028 (1.022, 1.037)*
Black (ref. white)	0.619 (0.536, 0.711)*	0.623 (0.541, 0.716)*	0.618 (0.534, 0.712)*
Other	1.009 (0.847, 1.203)	1.015 (0.850, 1.209)	1.012 (0.849, 1.212)
Female	1.412 (1.228, 1.624)*	1.428 (1.245, 1.640)*	1.423 (1.242, 1.637)*
Smoker	1.508 (1.27, 1.788)*	1.510 (1.274, 1.791)*	1.516 (1.278, 1.802)*
Body Mass Index	1.001 (0.993, 1.01)	1.001 (0.993, 1.009)	1.001 (0.993, 1.009)
ABI < 0.9	1.332 (1.146, 1.547)*	1.332 (1.143, 1.554)*	1.339 (1.146, 1.567)*
ACEI/ARB use	0.916 (0.799, 1.047)	0.916 (0.800, 1.052)	0.914 (0.798, 1.045)
Glucose (mg/dL)	1.000 (0.998, 1.001)	1.000 (0.998, 1.001)	1.000 (0.998, 1.001)
Hemoglobin A1c (%)	1.093 (1.037, 1.153)*	1.092 (1.036, 1.151)*	1.093 (1.036, 1.151)*
Hemoglobin (g/dL)	0.918 (0.88, 0.954)*	0.919 (0.883, 0.955)*	0.918 (0.880, 0.955)*
Calcium (mg/dL)	0.793 (0.699, 0.895)*	0.789 (0.697, 0.890)*	0.790 (0.698, 0.890)*
Creatinine	9.272 (7.973, 10.946)*	9.281 (7.933, 11.012)*	9.431 (7.973, 11.28)*
High BP	0.985 (0.824, 1.171)	0.990 (0.828, 1.183)	0.986 (0.826, 1.176)
SBP (mmHg)	1.008 (1.004, 1.013)*	1.008 (1.004, 1.013)*	1.008 (1.004, 1.013)*
DPB (mmHg)	0.999 (0.993, 1.006)	0.999 (0.993, 1.006)	1.000 (0.993, 1.006)
Diabetes	1.209 (1.036, 1.413)*	1.219 (1.041, 1.426)*	1.214 (1.037, 1.422)*
Diabetes - ϕ_{11}	-		
Diabetes - ϕ_{12}	-		
Diabetes - ϕ_{13}	-		
Hypertension	1.315 (1.062, 1.644)*	1.359 (1.096, 1.685)*	1.343 (1.087, 1.665)*
Hypertension - ϕ_{21}	-		
Hypertension - ϕ_{22}	-		
Hypertension - ϕ_{23}	-		
CVD	1.822 (1.605, 2.071)*	1.831 (1.606, 2.088)*	1.842 (1.623, 2.106)*
CVD - ϕ_{31}	-	-	-
CVD - ϕ_{32}	-	-	-
CVD - ϕ_{33}	-	-	-
α_1	-	-	-
α_2	-	-	-

*95% credible interval (CI) does not include hazard ratio (HR) of 1;
angiotensin-converting enzyme inhibitor(ACEI); angiotensin receptor blocker (ARB); blood pressure (BP); cardiovascular disease (CVD); hemoglobin A1c (HbA1c); systolic and diastolic BP (SBP, DBP)

Table S8: Sensitivity analyses. (i) Estimates of associations/linkage among trivariate outcomes: (A) longitudinal estimated glomerular filtration rate (eGFR), (B) recurrent cardiovascular (CV) events, and (C) terminal event (kidney failure or death), and (ii) model variance components. Sensitivity analyses with inverse gamma IG(1, 0.05) and IG(1, 0.5) compared to the main results reported using IG(1, 0.005).

**(i) Association Parameters**		**Estimate (95% CI)**	
	**IG(1, 0.005)**	**IG(1, 0.05)**	**IG(1, 0.5)**
eGFR/Recurrent			
η_{r0}	0.021 (0.010, 0.031)*	0.021 (0.001, 0.032)*	0.021 (0.001, 0.031)*
η_{r1}	-0.256 (-0.287, -0.227)*	-0.256 (-0.286, -0.226)*	-0.256 (-0.287, -0.226)*
eGFR/Terminal			
η_{t0}	0.007 (-0.006, 0.022)	0.007 (-0.007, 0.021)	0.008 (-0.005, 0.023)
η_{t1}	-0.698 (-0.756, -0.642)*	-0.696 (-0.761, -0.637)*	-0.706 (-0.775, -0.646)*
Recurrent/Terminal			
ζ	0.997 (0.808, 1.226)*	1.000 (0.795, 1.233)*	1.037 (0.823, 1.281)*
**(ii) Variance Components**			
σ_{b0}^2	35.002 (33.082, 37.030)*	35.021 (33.082, 37.111)*	35.021 (33.101, 37.083)*
σ_{b1}^2	5.300 (4.963, 5.670)*	5.301 (4.944, 5.671)*	5.311 (4.962, 5.694)*
ρ_{01}	0.308 (0.300, 0.327)*	0.308 (0.300, 0.326)*	0.308 (0.300, 0.326)*
σ_{ϵ}^2	37.782 (37.140, 38.432)*	37.780 (37.131, 38.432)*	37.781 (37.122, 38.443)*
σ_{ν}^2	0.567 (0.446, 0.692)*	0.566 (0.437, 0.710)*	0.552 (0.432, 0.685)*

*95% credible interval (CI) does not include estimate of 0

Table S9: Simple analyses of outcomes separately: (A) longitudinal estimated glomerular filtration rate (eGFR), (B) recurrent cardiovascular (CV) events, and (C) terminal event (kidney failure or death). Effect sizes (estimates and hazard ratios [HRs]) are given for one unit change in covariates.

**Variable**	**(A) Longitudinal eGFR Estimate (95% CI)**	**(B) Recurrent CV Events HR (95% CI)**	**(C) Terminal Event HR (95% CI)**
Intercept	55.004 (53.589, 54.913)*	-	-
Time, γ	-1.339 (-1.415, -1.261)*	-	-
Age (years)	-0.299 (-0.323, -0.277)*	1.027 (1.021, 1.034)*	1.002 (0.997, 1.007)
Black (ref. white)	6.307 (5.856, 6.778)*	0.966 (0.869, 1.067)	0.884 (0.803, 0.974)*
Other	1.040 (0.315, 1.684)*	0.888 (0.773, 1.012)	1.201 (1.065, 1.358)*
Female	-9.743 (-10.227, -9.215)*	0.874 (0.788, 0.968)*	1.038 (0.946, 1.143)
Smoker	-0.384 (-1.012, 0.222)	1.261 (1.112, 1.439)*	1.550 (1.384, 1.744)*
Body Mass Index	0.013 (-0.017, 0.042)	1.015 (1.009, 1.022)*	1.000 (0.994, 1.006)
ABI $<$ 0.9	-0.059 (-0.655, 0.543)	1.261 (1.130, 1.413)*	1.145 (1.025, 1.270)*
ACEI/ARB use	-1.047 (-1.506, -0.568)*	0.957 (0.865, 1.059)	0.960 (0.872, 1.054)
Glucose (mg/dL)	0.007 (0.002, 0.013)*	1.000 (0.999, 1.001)	1.000 (0.999, 1.001)
Hemoglobin A1c (%)	-0.27 (-0.472, -0.068)*	1.134 (1.088, 1.185)*	1.112 (1.074, 1.153)*
Hemoglobin (g/dL)	0.218 (0.091, 0.365)*	0.958 (0.928, 0.989)*	0.930 (0.903, 0.957)*
Calcium (mg/dL)	1.022 (0.554, 1.447)*	0.821 (0.748, 0.900)*	0.751 (0.689, 0.819)*
Creatinine	-25.538 (-25.966, -25.135)*	1.241 (1.131, 1.369)*	3.152 (2.927, 3.387)*
High BP	-0.459 (-1.126, 0.134)	1.006 (0.890, 1.1380)	1.077 (0.964, 1.212)
SBP (mmHg)	-0.035 (-0.05, -0.020)*	1.005 (1.002, 1.009)*	1.013 (1.01, 1.016)*
DPB (mmHg)	0.016 (-0.006, 0.037)	0.996 (0.991, 1.001)	0.997 (0.993, 1.002)
Diabetes	0.051 (-0.561, 0.589)	-	1.228 (1.097, 1.370)*
Diabetes - ϕ_{11}	-	1.195 (1.042, 1.376)*	-
Diabetes - ϕ_{12}	-	1.018 (0.833, 1.235)	-
Diabetes - ϕ_{13}	-	0.832 (0.658, 1.055)	-
Hypertension	-1.840 (-2.438, -1.226)*	-	1.314 (1.121, 1.557)*
Hypertension - ϕ_{21}	-	1.654 (1.346, 2.050)*	-
Hypertension - ϕ_{22}	-	1.166 (0.831, 1.692)	-
Hypertension - ϕ_{23}	-	0.936 (0.720, 1.640)	-
CVD	-0.567 (-1.040, -0.105)*	-	1.523 (1.394, 1.662)*
CVD - ϕ_{31}	-	2.675 (2.375, 3.031)*	-
CVD - ϕ_{32}	-	1.747 (1.467, 2.081)*	-
CVD - ϕ_{33}	-	1.713 (1.359, 2.162)*	-
α_1	-	5.259 (3.582, 7.721)*	-
α_2	-	1.747 (1.019, 2.948)*	-

*95% credible interval (CI) does not include estimate of 0 or hazard ratio (HR) of 1;
angiotensin-converting enzyme inhibitor(ACEI); angiotensin receptor blocker (ARB); blood pressure (BP); cardiovascular disease (CVD); hemoglobin A1c (HbA1c); systolic and diastolic BP (SBP, DBP)

Table S10: Additional simulation results for (A) lower and (B) higher variance of frailty terms compared to the main simulation studies. Also, additional simulation studies with (C) lower correlation of 0.3. Simulations provided are for $n = 4000$, and censoring rate 50%, and 45% recurrent events. Given are bias, mean squared error (MSE), and average coverage probabilities (CP) of the 95% credible interval averaged over 300 datasets.

	(A) Lower variance				(B) Higher variance				(C) Lower correlation			
	True	Bias	MSE	CP	True	Bias	MSE	CP	True	Bias	MSE	CP
Longitudinal												
β_{0l}	47.00	0.002	0.002	95.8	47.00	0.001	0.002	95.7	47.00	0.002	0.002	95.8
β_{1l}	0.60	0.001	3.5×10^{-4}	95.3	0.60	-0.001	3.3×10^{-4}	95.1	0.60	-0.001	3.8×10^{-4}	95.5
ϕ_l	-1.30	-0.002	0.001	95.1	-1.30	-0.001	0.003	95.6	-1.30	-0.001	0.002	95.1
γ	-1.50	-0.002	0.001	95.4	-1.50	-0.002	0.001	96.9	-1.50	-0.003	0.001	95.1
Recurrent Events												
β_r	0.20	0.010	0.001	95.1	0.20	0.008	0.001	95.6	0.20	0.008	0.001	95.2
ϕ_1	1.04	0.003	0.004	95.1	1.04	0.001	0.008	95.6	1.04	0.002	0.005	96.0
ϕ_2	0.93	0.011	0.013	95.2	0.93	0.012	0.011	95.9	0.93	0.015	0.007	95.1
ϕ_3	0.79	0.019	0.022	96.5	0.79	0.013	0.012	96.6	0.79	0.017	0.020	94.9
α_1	0.80	0.026	0.016	93.8	0.80	0.024	0.006	94.9	0.80	0.028	0.015	94.6
α_2	0.53	0.015	0.018	95.9	0.53	0.009	0.019	95.4	0.53	0.019	0.023	95.1
η_{r0}	0.60	0.015	0.003	93.9	0.60	0.009	0.002	95.4	0.60	0.014	0.005	94.8
η_{r1}	0.60	0.003	0.007	95.9	0.60	0.002	0.003	96.7	0.60	0.008	0.006	95.0
Terminal Event												
β_t	0.50	0.018	0.002	94.5	0.50	0.014	0.003	95.5	0.50	0.014	0.002	94.8
ϕ_t	0.45	0.012	0.007	95.6	0.45	0.005	0.011	96.1	0.45	0.005	0.007	95.5
η_{t0}	0.90	0.027	0.006	94.2	0.90	0.024	0.004	94.4	0.90	0.035	0.012	93.1
η_{t1}	0.90	0.016	0.016	95.2	0.90	0.013	0.008	95.7	0.90	0.019	0.013	94.5
ζ	1.20	0.044	0.018	94.6	1.20	0.040	0.007	95.2	1.20	0.047	0.015	94.4
Variance/Correlation												
$\sigma^2_{b_0}$	1.00	0.002	0.001	95.0	2.00	-0.002	3.6×10^{-4}	95.6	1.24	-0.003	0.001	95.1
$\sigma^2_{b_1}$	0.50	0.012	0.001	93.2	1.50	-0.010	0.001	93.0	0.80	-0.015	0.003	93.3
ρ_{01}	0.50	0.025	0.002	95.4	0.50	0.018	3.7×10^{-5}	96.8	**0.30**	0.026	0.002	95.0
σ^2_{ε}	1.32	0.001	1.2×10^{-5}	96.0	1.32	0.001	1.2×10^{-5}	96.9	1.32	0.003	2.2×10^{-5}	95.5
σ^2_{ν}	1.20	-0.008	0.002	95.5	2.00	-0.005	0.003	95.5	1.44	-0.009	0.002	95.4

Table S11: Additional simulation results for Gompertz and log-normal baseline hazard settings with $n = 4000$, censoring rate 50%, and 45% recurrent events. Given are bias, mean squared error (MSE), and average coverage probabilities (CP) of the 95% credible interval averaged over 300 datasets.

	Gompertz				Log-normal			
	True	Bias	MSE	CP	True	Bias	MSE	CP
Longitudinal								
β_{0l}	47.00	0.001	0.002	95.7	47.00	0.003	0.002	96.3
β_{1l}	0.60	0.001	3.7×10^{-4}	95.0	0.60	0.001	3.8×10^{-4}	94.9
ϕ_l	-1.30	-0.002	0.002	95.6	-1.30	-0.002	0.001	95.7
γ	-1.50	-0.002	0.001	96.4	-1.50	-0.001	0.001	95.8
Recurrent Events								
β_r	0.20	0.005	0.001	95.7	0.20	0.006	0.001	95.4
ϕ_1	1.04	-0.001	0.005	95.2	1.04	-0.002	0.006	95.4
ϕ_2	0.93	-0.013	0.014	95.6	0.93	0.011	0.016	95.9
ϕ_3	0.79	0.017	0.021	96.0	0.79	0.016	0.020	96.5
α_1	0.80	-0.027	0.015	94.2	0.80	0.028	0.015	94.9
α_2	0.53	-0.013	0.020	95.6	0.53	0.019	0.022	95.9
η_{r0}	0.60	-0.008	0.003	95.5	0.60	0.011	0.002	95.0
η_{r1}	0.60	0.003	0.008	95.7	0.60	-0.004	0.005	95.2
Terminal Event								
β_t	0.50	-0.011	0.002	94.7	0.50	0.015	0.002	95.2
ϕ_t	0.45	-0.005	0.007	95.7	0.45	-0.007	0.007	96.1
η_{t0}	0.90	-0.025	0.005	94.7	0.90	-0.023	0.005	95.0
η_{t1}	0.90	0.018	0.013	95.7	0.90	0.014	0.010	95.2
ζ	1.20	-0.045	0.014	95.5	1.20	-0.042	0.012	95.0
Variance/Correlation								
$\sigma_{b_0}^2$	1.24	-0.002	2.0×10^{-4}	95.7	1.24	-0.003	2.1×10^{-4}	95.4
$\sigma_{b_1}^2$	0.80	-0.007	0.001	94.0	0.80	-0.008	0.001	93.9
ρ_{01}	0.50	0.024	0.001	95.7	0.50	0.024	0.001	95.5
σ_{ε}^2	1.32	0.001	1.2×10^{-5}	96.2	1.32	0.001	1.1×10^{-5}	96.1
σ_{ν}^2	1.44	-0.004	0.003	95.6	1.44	0.007	0.005	95.5
